# Airway-On-A-Chip: Designs and Applications for Lung Repair and Disease

**DOI:** 10.3390/cells10071602

**Published:** 2021-06-26

**Authors:** Tanya J. Bennet, Avineet Randhawa, Jessica Hua, Karen C. Cheung

**Affiliations:** 1 School of Biomedical Engineering, University of British Columbia, Vancouver, BC V6T 1Z4, Canada; tanyabennet@ece.ubc.ca (T.J.B.); avineet.randhawa@ubc.ca (A.R.); jessica.hua@ubc.ca (J.H.); 2 Centre for Blood Research, University of British Columbia, Vancouver, BC V6T 1Z4, Canada; 3 Department of Electrical & Computer Engineering, University of British Columbia, Vancouver, BC V6T 1Z4, Canada

**Keywords:** airway-on-a-chip, microfluidics, extracellular matrix, epithelial cells, endothelial cells, fibroblasts, remodeling, exposures, cigarette smoke, electronic cigarettes, analysis methods, bio-imaging

## Abstract

The lungs are affected by illnesses including asthma, chronic obstructive pulmonary disease, and infections such as influenza and SARS-CoV-2. Physiologically relevant models for respiratory conditions will be essential for new drug development. The composition and structure of the lung extracellular matrix (ECM) plays a major role in the function of the lung tissue and cells. Lung-on-chip models have been developed to address some of the limitations of current two-dimensional in vitro models. In this review, we describe various ECM substitutes utilized for modeling the respiratory system. We explore the application of lung-on-chip models to the study of cigarette smoke and electronic cigarette vapor. We discuss the challenges and opportunities related to model characterization with an emphasis on in situ characterization methods, both established and emerging. We discuss how further advancements in the field, through the incorporation of interstitial cells and ECM, have the potential to provide an effective tool for interrogating lung biology and disease, especially the mechanisms that involve the interstitial elements.

## 1. Introduction

The lung has a complex multicellular 3-dimensional (3D) architecture in which cells interact with each other, the surrounding extracellular matrix (ECM), and the external environment. These interactions are crucial for the development, maintenance, and regulation of the structures and functions of the lung [1]. In general, the respiratory system can be separated into two zones: the conducting and respiratory zones. The conducting airways (trachea to terminal bronchioles) provide a passageway for air to move into and out of the lungs, while the respiratory airways (respiratory bronchioles, alveolar ducts, and alveoli) are where gas exchange occurs. The conducting airways play a major role in the response to inhaled environmental particulates and are a major site of pathology in respiratory disorders, including asthma and chronic obstructive pulmonary disease (COPD) [2,3].

In humans, the conducting and respiratory airways are lined with a highly specialized epithelium that interfaces with both the internal microenvironment and the external environment. The apical surface of the epithelium is exposed to air and inhaled substances, while the basal surface is in contact with the underlying submucosa (ECM, interstitial cells, and vasculature). The unique air–liquid interface (ALI) present in the airways results in the epithelium acting as the first line of defense against inhaled particulates, toxins, and pathogens [4,5]. The complex architecture of the lung, including and especially the ALI, is challenging to replicate in vitro. Currently, widely used in vitro models simplify the microarchitecture, cellular composition, ECM, and/or microenvironment. These simplifications limit the ability to replicate complex interactions between cells, the extracellular matrix, and the microenvironment that are necessary to obtain organ function and investigate airway biology.

Microfluidic lung-on-a-chip models have emerged as tools that have the potential to replicate the microarchitecture, microenvironment and interactions present in the in vivo setting [6]. Lung-on-a-chip (and other organ-on-a-chip) devices are based on the science and technology of microfluidics, stemming from the concept of “lab-on-a-chip”. Adapting from microchips, the “lab-on-a-chip” sought to miniaturize lab experiments by applying microfluidics, where microchannels can be used to process or manipulate small volumes (generally, 10^−9^ to 10^−18^ L) [7,8,9]. Appealing benefits of microfluidic technology, including the usage of low and controllable volumes, rapid mixing speeds and responses, and precise control of physical and chemical properties [10,11], have led to the development of “chip” models as biomimetic platforms with controllable parameters such as concentration gradients, shear force, and cellular or tissue architecture [9]. Although various lung-on-a-chip devices have been developed, many focus on replicating the alveoli. In order to study lung biology and disease pathologies that center around the submucosa layer of the lungs, a need still exists within the lung-on-a-chip field to develop models that incorporate cell types that are found in the submucosa layer. Expansion into this space will also enhance the ability to study the complex cellular events that occur in response to inhaled agents prior to reaching the site of gas exchange, and further investigate the pathogenesis of airway diseases that occur upstream of the alveoli. The focus of this review is to review and evaluate current lung-on-a-chip technologies suitable for studying airway remodeling and repair, provide an example of their application in the study of cigarette smoke and electronic cigarette vapor, as well as sharing our perspective of advancements required to effectively harness these tools.

## 2. Modeling Lung Biology On-Chip

**Figure 1:**
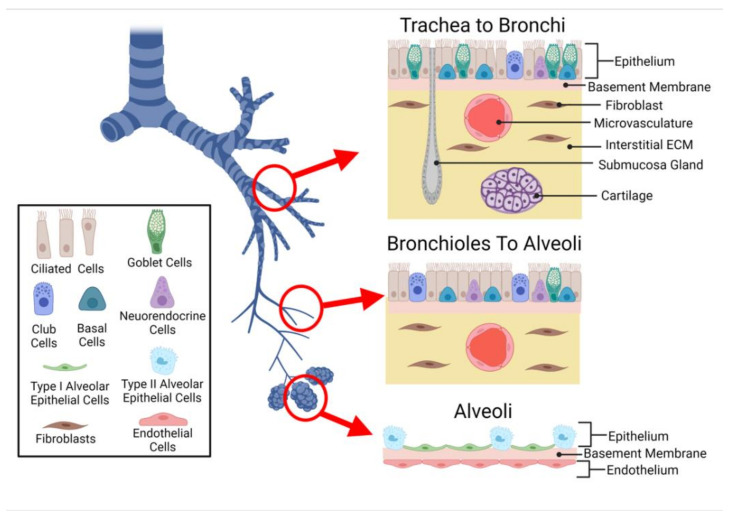
Cells of the Lung. The conducting airways are lined with a pseudostratified epithelium, including mucus producing goblet cells, ciliated cells, neuroendocrine cells, and basal cells. The alveolar epithelium is composed of type 1 flattened epithelial cells and cuboidal type 2 epithelial cells. The submucosa layer of the airways contains interstitial and vascular cells, while the alveolar–capillary interface sees the epithelium in close proximity to the endothelium.

To facilitate each region’s primary function, the cellular composition, structure, and spatial organization of cells differ throughout the respiratory system (Figure 1).

### 2.1. Cellular Composition

Previous reviews [12,13,14] have highlighted the importance of selecting appropriate cells when creating an in vitro model. The cell selection process is often the starting point for model development, as cells form the basis of the model. In general, the main goal of the cellular input into an in vitro model should be to incorporate the most important cell phenotypes and culture them in a microenvironment that facilitates more natural cell-to-cell interactions. The lung is made up of approximately 40 different cell types [15]. The multicellular nature of the lung in combination with the diversity of cells, interactions, interfaces, functions, and morphology throughout the respiratory system make it a difficult system to model. With such diversity it is therefore important that the cell phenotype(s), origin, and microarchitecture incorporated into an in vitro model reflect the region of interest and consider the most relevant cell types. It is also important that during the development and selection process, the desired endpoints are carefully considered as they may dictate the level of complexity attainable.

The benefits and limitations associated with cell origin have been extensively covered in review papers [13,14,16]. Although both human cell lines [17,18,19,20,21,22] and primary cells derived from both healthy [23,24,25,26,27,28], and diseased donors [23,29] have been used to create a diverse range of in vitro lung-on-a-chip models, a common strategy employed is to focus on the epithelium first and then add complexity by incorporating additional cell populations to mimic multicellular structures, establish physiological interfaces, and capture desired cell–cell interaction. Models containing primary cells are more desirable than those containing cell lines, as they are able to obtain and retain in vivo-like phenotypes of the airways [23,24,27,28].

Although currently not used in lung-on-a-chip technology, integrating human-induced pluripotent stem cells (hiPSC) could provide the opportunity to further study lung development, respiratory diseases, as well as offer a platform for personalized medicine [16].

#### 2.1.1. Epithelium

The epithelium operates as a physical barrier and plays a critical role in fluid balance, immune response, and tissue repair [4,30,31]. Although the general function of the epithelium remains constant throughout the respiratory tract, the specific cellular composition is location-dependent: the epithelium transitions from pseudostratified, ciliated, columnar morphology present in the proximal regions of the conducting zone to simple cuboidal in the small airway, then into a simple squamous epithelium in the alveoli [32].

Depending on location, the epithelium is composed of several cell types including ciliated, basal, goblet, and club cells [33,34,35]. The cells of the epithelium work together to provide a number of defense mechanisms against inhaled particulates, including tight junctions, mucus production, mucociliary clearance, and secretion of molecules and mediators to signal to other cells of the lung (i.e., fibroblasts, immune cells) [34,35,36]. The main role of ciliated cells is to help physically remove debris and inhaled pathogens. This is accomplished via the mucociliary elevator and apically secreted factors, such as metabolic enzymes [37]. Goblet cells are responsible for secreting mucus, which helps trap inhaled particulates and pathogens [36]. Club cells are also secretory in nature, and increase in number, inversely to the number of goblet cells to ensure maintenance of protection. Club cells also work with Basal cells to regenerate the epithelium after injury [35]. Lung-on-a-chip devices composed of primary airway epithelial cells have been shown to recapitulate physiological cell compositions, consisting of mucus producing goblet cells and beating ciliated cells [22,23,24,28]. In respiratory diseases such as Chronic Obstructive Pulmonary Disease, Goblet cell hyperplasia results in excess production and secretion of mucus, while cilia dysfunction compromises clearance [35,37]. Lung-on-a-chip devices have been shown to enable the observation of mucus secretion which can be difficult in standard Transwell^®^ (Corning, Corning, NY, USA USA) in vitro models, as well as recapitulate hypersecretion observed in many lung diseases (i.e., asthma and COPD) [23,28]. Mucociliary transport is another aspect of airway biology that lung-on-a-chip devices can replicate in a more physiological manner when compared to standard in vitro models. Benam et al. demonstrated how in microfluidic devices, the microchannel design combined with inlets and outlets enables the coordinated movement of cilia to move particles in a manner that reflects what is observed in healthy human lung airways [23].

Unlike other parts of the respiratory tract, the alveoli contain a unique epithelium composed of two types of pneumocytes: Alveolar Type I and Type II. Type I cells are involved in gas exchange [38]. Type II cells play a major role in immune response and remodeling, as they can synthesize a variety of matrix/surfactant components including fibronectin, collagen IV, and laminin [39]. Although 95% of the alveolar epithelial barrier is composed of Type I cells, many cell-line-based alveoli lung-on-a-chip models are constructed from alveolar type II epithelial-like cell line (e.g., A549) [17,18,19,40].

#### 2.1.2. Submucosa

In the airways, the epithelium overlays the submucosa, composed of airway smooth muscle cells, fibroblasts, myofibroblast, pericytes, and neural cells. The connective tissue and interstitial cells provide structural and functional support. Fibroblasts are the most abundant cell type found in the lung interstitium, playing a crucial role in airway repair, remodeling, and inflammation. The primary function of lung fibroblasts is the production of ECM proteins (i.e., type I and III collagen, elastin, fibronectin, and proteoglycans) [41,42]. However, they also secrete metalloproteinases to trigger ECM degradation and inflammatory signals such as tumor necrosis factor- post injury [42]. Under various stimuli (i.e., mechanical cues, changes in microenvironment, cellular communication), lung fibroblasts undergo migration, proliferation, activation, and differentiate into contractive myofibroblasts [43]. The presence of myofibroblasts is a key hallmark of many respiratory diseases as they are associated with increased ECM deposition and the development of fibrotic tissue [42].

Just as the epithelium changes throughout the respiratory tract, the submucosa differs within each region. As the large airways transition into the small airway the submucosa begins to lack cartilage and submucosal glands. Within the alveoli the submucosa is absent, with only a thin non-cellular basement membrane separating the epithelium from the endothelium to create the alveolar–capillary barrier.

#### 2.1.3. Lung Microvasculature

The submucosa also contains a network of blood vessels known as the lung microvasculature that supports lung tissues by supplying it with oxygen and nutrients, while removing metabolic wastes via circulation. The inner walls of the microvasculature are lined with a monolayer of endothelial cells resting on the basement membrane that separates circulating blood from the surrounding tissue and operates as a semi-selective barrier [44]. As the endothelium is in direct contact with blood (including the circulating cells found in blood), the endothelial barrier regulates the passage of compounds, fluids, and pathogens into interstitial tissues, playing an important role in gas exchange, molecular transport, immune response, and fluid balance. In the alveoli, endothelial and epithelial layers are only separated by a thin basement membrane (alveolar–capillary barrier), enhancing gas exchange.

In vivo, circulating blood exerts shear stress on the endothelium as the blood flows through the vasculature and past the cell surface. The microvascular wall shear stress experienced by the endothelial cells ranges from 1 to 10 dyn/cm^2^, dependent upon the microenvironment [45]. Shear stress modulates various behaviors including morphology, proliferation, differentiation, and communication, aiding in barrier formation [44]. Microfluidic organ-on-a-chip devices manipulate fluid flow to recapitulate the dynamic mechanical forces that endothelial cells experience in vivo [26].

In addition to nutrient supply, the vasculature also plays a major role in immune response as immune cells (e.g., neutrophils) circulate through the blood vessels. Lung-on-a-chip devices have leveraged their microfabricated design and features to replicate how lung microvasculature supports neutrophil-mediated host defense by introducing neutrophils into the vascular compartment, and monitoring migration into the air channel in the presence of foreign materials [26].

The diversity of the cellular combination throughout the regions of the respiratory tract highlights the importance of creating models with appropriate cells, as well as why alveoli models are limited when used for investigating physiological and pathologic responses associated with other regions of the respiratory tract. Current lung-on-a-chip devices have shown that in vivo region-specific phenotypes can be obtained by using human primary cells from the desired region, even if the construction of the microfluidic device remains the same. For example, Benam et al. modified the alveolus-on-a-chip developed by Huh et al. to represent the small airway, by replacing alveolar epithelial cells with small airway epithelial cells, to obtain an epithelium that reflected the region of interest [23,26].

### 2.2. Interfaces

**Figure 2:**
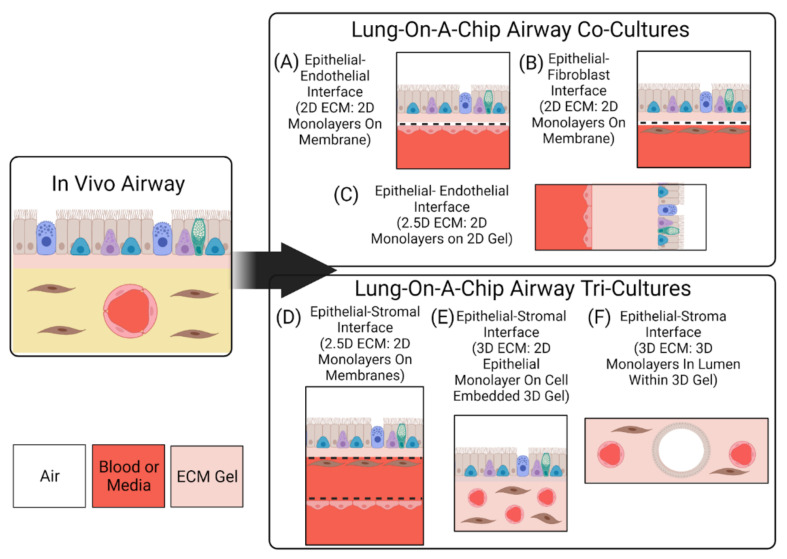
Interfaces and Dimensions of Lung-On-A-Chip Devices. Lung-on-a-chip devices can be made to incorporate monoculture, co-culture, or tri-culture of cells. Here, 2D, 2.5D, and 3D refer to the organization of the cell layers on the supporting scaffolds. Monocultures (not shown) would comprise only the epithelial layer as in (**A**). (**A**) Epithelial cell monolayer on the apical side of a permeable membrane, with endothelial cell monolayer on the basal side of the membrane. (**B**) Epithelial cell monolayer on the apical side of a permeable membrane, with fibroblast cell monolayer on the basal side of the membrane. In (**A**,**B**), the monolayers are stacked vertically, i.e., parallel to the objective lens’s optical axis in microscopy. (**C**) The epithelial and endothelial cell monolayers are cultured on a hydrogel, and the layers are stacked perpendicular to the objective lens’s optical axis in microscopy. (**D**) The tri-culture features two permeable membranes, separating the stromal cells from the endothelial cells. (**E**) The tri-culture comprises an epithelial cell monolayer on top (apical side) of a 3D gel, inside which the stromal cells and vascular endothelium are embedded. (**F**) The tri-culture comprises cell-lined lumens within a 3D gel embedded with stromal cells. The epithelial cells form a single cell layer lining an air-filled lumen, while the endothelial cells form a single cell layer lining a blood/media-filled lumen.

To replicate morphology and functionality of the lung, it is not only important to capture cellular composition, but it is also essential to replicate physiological interfaces—tissue–tissue and air–liquid—to mimic the in vivo structures, as well as obtain an in vitro model that replicates in vivo phenotypes [12,46]. Most lung-on-a-chip devices have leveraged traditional micro-electrical-mechanical systems (MEMS) technology to create compartmentalized microstructures with physiological dimensions that mimic in vivo 3D spatial cell organization. The microfabricated features (often microchannels) enable living cells to be cultured in physiological dimensions, while the compartmentalization allows cells to be utilized as building blocks to create complex interfaces which mirror the native environment. Similar to traditional cell culture inserts, most lung-on-a-chip devices utilize a porous membrane to separate cellular compartments and create an ALI [18,19,21,24,26,47,48]. Porous membranes, combined with a compartmentalized design and microfluidics, allow for each cellular compartment to be independently seeded with cells, while facilitating nutrient exchange and intercellular cross-talk. This approach enables cells to be cultured on both sides of the membrane, enabling a model to construct tissue–tissue interfaces that replicate those present in vivo. The combination also offers the ability to precisely control the microenvironment created within the chips; the independent chambers enable the creation of unique microenvironments for the various cells. Many groups have taken advantage of the independent nature of their compartmentalized designs to create an ALI [18,19,20,21,22,24,25,26,27,28,40,47,49,50,51]. Figure 2 highlights the various ALI architectures that can be generated using microfabrication techniques.

The organization of the compartmentalized channels can be vertically stacked [19,22,23,24,26,50] or parallel [20,27]. However, the general aim is to have the airway and vascular components be separated by an ECM substitute, in a manner that replicates in vivo separation distances. The types of ECM substitutes and their evolution in lung-on-a-chip models is described later in this review. Recently, some microfluidic platforms have incorporated hydrogels in an attempt to further enhance the 3D nature of lung-on-a-chip models [22,27,28]. With this approach, cells are either: cultured on the gel’s surface, or embedded into the bulk of the structure. Cell-laden hydrogels provide the opportunity to arrange interstitial cells (i.e., fibroblasts) into a microenvironment that is more representative of the in vivo setting, and further enhance the 3D cell–cell and cell–ECM interactions.

To replicate the internal diameter of the small airways (<2 mm), Benam et al. designed the upper compartment of a vertically stacked, membrane separated chip to contain a 1 × 1 mm rectangular cross section [23]. The dimensions of the channel reflect the in vivo setting, however the rectangular geometry of the channel does not replicate the circular geometry of the luminal portion of the airway. The techniques employed in traditional microfabrication are normally limited to rectangular geometries, causing cells to be grown in flat 2D monolayers. To more accurately replicate the lungs from a structural and architectural perspective, microfluidic systems incorporating hydrogels have been shown to enable the creation of perfusable lumen structures [27,52]. Replicating the geometry of the airway lumen and blood vessel is important, as cells grown in lumen shaped monolayers are a more physiological representation and replicate in vivo phenotypes [53].

Hydrogel-containing microfluidic models also provide the opportunity to take advantage of the cells’ ability to self-assemble. For example, endothelial cells grown in ECM hydrogels have been shown to self-assemble into blood vessel-like structures [28,54]. Leveraging self-assembly within a lung-on-a-chip device has the potential to increase the physiological relevance of the model, but this can complicate analysis and decrease the control over the microenvironment. The decrease in control would also result in an increase in variability between samples.

Lung-on-a-chip devices can be operated in submerged [17,18,20] or ALI [19,25,27,28,55,56] conditions. Devices containing a submerged condition are less representative of the in vivo setting than those containing ALI, as they do not differentiation into an in vivo-like phenotype; for example, Lenz et al. have found that cells exposed to airborne zinc oxide nanoparticles in an ALI culture had higher transcript levels of pro-inflammatory markers, but similar viability levels, compared to cells exposed in submerged cultures [57]. The discrepancy from the in vivo environment can be linked to the fact that, although the device organizes lung-specific cells into a 3D stratified tissue configuration, the individual cell populations remain planar in structure as media is exposed to all sides of the cells. These 2D monolayers therefore limit the model’s ability to replicate the 3D nature of the in vivo environment and affect various cell behavior.

To replicate the 3D multicellular structure of the lung, an ALI can be established on chip using protocols similar to those used for traditional cell culture inserts. Cells in the apical compartment are cultured to confluence, then media is removed (“airlifted”) to polarize the epithelium and trigger differentiation towards a mucociliary phenotype. When an ALI is established within a microfluidic platform, epithelial cells become polarized as the apical surface of the epithelium is exposed to air while nutrients are supplied via the basolateral side. This configuration creates a better representation of the physiological environment of the lung, and cells cultured at ALI display more physiological phenotypes such as tight junctions, mucus production and cilia beating [23]. Blood flow can be recapitulated in lung-on-a-chip devices by perfusing the vascular compartments. To further replicate the vascular endothelium, endothelial cells can be seeded into the lower microchannel (or vessel structure) and flow rates can be adjusted to expose endothelial cells to physiological shear. These aspects further enhance the model’s ability to replicate complex cell–cell interactions.

The mechanical effect of breathing in the alveoli has been modeled in several microfluidic systems. The seminal work of Huh et al. showed that an organ-on-chip system could be used to apply cyclic mechanical strain to an alveolar–capillary model in a flat, rectangular geometry [26]. Stucki et al. created an alveolar barrier that could be stretched in three dimensions [50,56]. They used a micro-diaphragm to stretch a thin, porous alveolar barrier on which epithelial and endothelial cells were cultured, in order to model the cyclic strain in the alveoli during breathing. The stretchable PDMS membranes were integrated with a pneumatic component such that an electro-pneumatic pump would apply a negative pressure, resulting in 0.2 Hz cyclic stretch corresponding to 10% linear strain. More recently, that group replaced the PDMS membrane with a stretchable and biodegradable membrane comprising collagen and elastin, to better reproduce the physical properties of the alveolar basal membrane [51]. A thin gold mesh was used to support the hydrogel solution and cells could be cultured at an air–liquid interface for several weeks, and a vacuum system was used to apply cyclic strain to the membranes to mimic breathing. Pulmonary emphysema is a condition that can lead to over-inflation and damage to the alveoli. In emphysema, the alveolar barrier can be remodeled, and this biodegradable ECM will permit the study of this process. Huang et al. created a porous hydrogel comprising GelMA on which alveolar epithelial cells formed monolayers, because they found that 7% GelMA had a stiffness close to normal lung tissue [25]. Cyclic strain was applied to the alveolar sac-like structures at 0.2 Hz using a negative pressure system.

**Figure 3:**
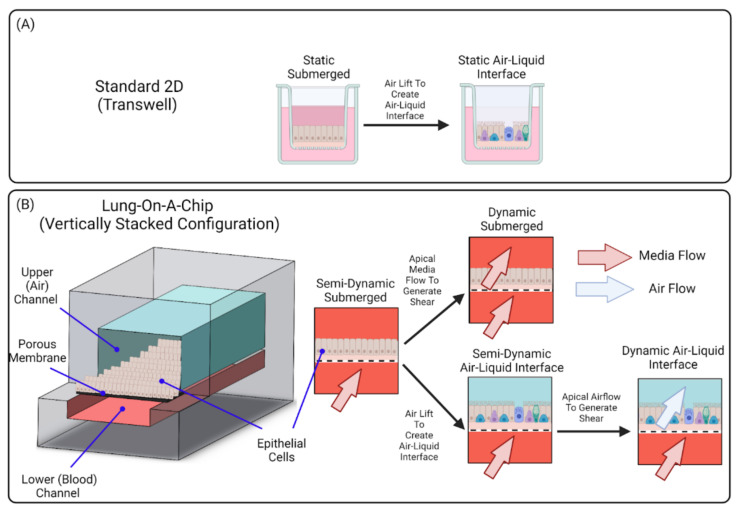
Air–Liquid Interface Configurations. (**A**) Standard 2D ALIs require simply removing media from the top compartment while (**B**) ALI in lung-on-a-chip devices can be operated in semi-dynamic or dynamic mode. Lung-on-a-chip platforms permit recapitulation of the flow physiology, both the blood flow in the basal compartment, as well as air flows. Lung-on-a-chip platforms can also mimic the submucosal layer. Finally, lung-on-a-chip models permit delivery of aerosols to model inhalation of droplets or particulate matter.

In vivo, the luminal portion of the airways experience shear stress (0.5 to 3 dyn/cm^2^) as air flows into and out of the lung [58]. Although compartmentalization provides the opportunity to replicate this feature of the airways, many groups do not incorporate airflow into their airway-based models [22,24,27,28]. As these models do incorporate flow in the vasculature chamber, they are more representative of the in vivo environment than traditional static Transwell^®^ models. However, because the air microenvironment remains static it precludes recapitulation of the full dynamic nature of the in vivo setting. Therefore, these models are more representative of a semi-dynamic ALI. Figure 3 shows a visual representation of the various conditions that can be established on lung-on-a-chip devices. Lung-on-a chip devices containing semi-dynamic ALIs have been shown to replicate physiological phenotypes, however incorporating additional support systems (i.e., compressed air supply) that can follow air through the air channel has the potential to further enhance the model’s ability to mimic inhalation mechanisms and investigate interactions between the external and internal environments. Once established, a dynamic ALI can also be utilized to conduct exposure studies in a more physiologically and clinically relevant context [6]. Benam et al. created a custom smoking machine to replicate biomimetic exposure, however their non-exposed systems remain in a semi-dynamic state [59].

### 2.3. Interactions

The lung microenvironment is dynamic, with cells interacting with each other through direct (cell-to-cell contact) and indirect (paracrine signaling) means. To replicate the heterotypic cell–cell interactions in vitro, it is important that the model incorporates various cell types found in lung tissue, including epithelial cells, fibroblasts, vascular endothelial cells, and immune cells. In the case of disease modeling, it is also important to construct lung-on-a-chip models from healthy and disease donors. Constructing lung-on-a-chip devices from cells obtained from donors with respiratory diseases enables recapitulation of in vivo disease characteristics. Benam et al. demonstrated that an airway-on-a-chip device composed from COPD patient epithelial cells can reconstitute goblet cell hyperplasia, ciliary dysfunction, cytokine hypersecretion, infection-based exacerbation, and smoke-induced pathologies (i.e., oxidative stress) [23,60]. Benam et al. also highlighted the unique “personalized” potential of lung-on-a-chip technologies by creating devices composed of cells obtained from the same patient and culturing them with or without exposure to cigarette smoke to enable the study of patient specific responses [60].

To create a protective barrier between the external environment and sub-epithelial components, epithelial cells form sheets with strong cell-to-cell attachments in the form of protein complexes (i.e., tight, gap, and adherens junctions). Cell-to-cell contacts are essential for polarization and direct cell signaling between adjacent epithelial cells [61]. When cultured in single cell populations at ALI, epithelial cells in lung-on-a-chip devices can exhibit polarized morphology and expression of tight junctions [18,25]. The ability to capture these in vivo phenotypes indicates that simple models can be used for preliminary permeability and toxicity studies. However, due to the lack of additional cell types, these simplified models are limited in their ability to replicate indirect cell-to-cell interactions and cross-talk.

Once an in vivo-like epithelium is established, complexity and relevance of the model for investigating more complex biological interactions can be achieved by co-culturing with mesenchymal, vascular, neural, or immune cells. In vivo, cells communicate with themselves and other cells through signaling to induce and modulate functions and responses. Since cells in the body do not exist in isolation, co-culturing cells can provide a more representative microenvironment.

In the alveoli, the epithelium and surrounding capillaries have an intimate relationship due to the close proximity of cells. Although not in direct contact, the extremely thin basement membrane results in many interactions. Since the alveoli can be seen as the functional unit of the lungs, it has been extensively modeled using microfluidic organ-on-a-chip platforms [17,18,19,20,21,25,26,40,47,50,51,56]. Co-cultures of alveolar epithelial and endothelial cells at ALI on-chip have shown to exhibit in vivo phenotypes and functions [20,26,50,56]. The two cell types are seeded on opposite sides of a porous membrane and then an ALI is established by exposing the upper (epithelial) channel to air, while exposing the lower (endothelium) channel to fluid flow to support cells and replicate physiological shear. Xu et al. showed the impact of co-culturing epithelial and endothelial cells both in isolation and coupled with perfusion. When in monoculture format, perfusion of the vascular channel had no effect on permeability, but when co-cultured with endothelial cells not only did the co-culturing improve barrier function but the addition of flow increased it further emphasizing the importance of compounding elements [62]. These results also highlight the importance of incorporating flow and although alternative in vitro models such as organoids have been shown to recapitulate airway phenotypes, they lack blood flow and fluid shear conditions. Additional mechanical cues have also been incorporated into lung-on-a-chip devices to mimic the cyclic strain that occurs at the alveolar–capillary interface [21,26,56]. Incorporating this dynamic feature can allow lung-on-a-chip devices to be used to gain insights to the cell–cell interactions and responses linked to these mechanical cues.

To investigate the cell–cell interactions that occur in the airways, Benam et al. co-cultured bronchiolar epithelial cells and microvascular endothelial cells using a modified version of the chip developed by [26] to create a differentiated epithelial–endothelial interface that replicates the small airways [23]. The model utilized cells from the region of interest, however the interstitial compartment of the small airways was simplified to a 2D porous membrane limiting cellular interactions to 2D.

Since the lung acts as a physical barrier to pathogens it generates a strong immune response. Replication of this immune response in vitro depends on incorporating the cells, interfaces, ECM, and mechanical cues that create the dynamic cell–cell interactions. In the instance where certain cell types are not incorporated into a model, a response to a specific mediator can be simulated by introducing cytokines, chemokines, or growth factors into a specific channel and observing the response [23]. In compartmentalized lung-on-a-chip devices, the independent access to channels allows for heterotypic cell interactions by paracrine signaling across the ECM substitute to be investigated.

Incorporating the microvasculature into a lung-on-a-chip device is not only important to support cell culture and provide physiological shear forces to the cells, but it is also essential for enhancing a model’s suitability for disease modeling and investigating immune response. To replicate the interactions that exist between cells of the lung and circulating immune cells in the blood, immune cells (e.g., neutrophils) can be introduced into the vasculature channel via perfusion. For instance, a model that recapitulates viral infection of asthmatic airway epithelium was established by infecting a fully differentiated human mucociliary airway epithelium to human rhinovirus 16, then stimulating it with IL-13 [48].

Although respiratory diseases afflict more than just the alveoli, other regions of the respiratory tract have been modeled to a lesser extent. This may be due to the increase in complexity in creating a lung-on-a-chip model to reproduce aspects of the in vivo lung that incorporate more cell phenotypes, microarchitectures, and interactions such as those which include the interstitial ECM and microvasculature. For example, in Zamprogno et al.’s model of the air–blood barrier, the histological arrangement and cellular heterogeneity of the alveoli is well captured. They developed a model consisting of multiple stretchable alveoli with differentiated epithelium, both type I and type II, cultured atop of a thin ECM basement membrane with primary endothelial cells on the basal side [51]. However, other lung-on-a-chip models intended to reproduce regions such as those with interstitial ECM still require appropriate cell types such as fibroblasts and correct ECM constituents, or regions with luminal vessels are still limited by their rectangular geometry. Recent trends in the development of lung-on-a-chip models, such as the incorporation of a 3D hydrogel, indicate progress towards reproducing more accurate histological arrangements and including further cellular heterogeneity.

In vivo, fibroblasts actively participate in airway repair and remodeling via deposition and degradation of the extracellular matrix, as well as interactions with other cells including epithelial cells and immune cells. Fibroblasts are heavily influenced by autocrine and paracrine signaling and can be stimulated by cytokines, growth factors (i.e., transforming growth factor beta), cell–cell communication, and cell–ECM interactions [63,64]. In the case of epithelial injury, epithelial cells trigger the migration, proliferation, activation, and differentiation of fibroblasts. This results in the accumulation of fibroblasts and ECM at the site of injury [43]. When controlled, this response is effective for tissue repair however, excessive accumulation can result in abnormal tissue function. Fibroblast also secrete mediators that can interact with epithelial and endothelial cells. In respiratory diseases such as chronic obstructive pulmonary disease (COPD), epithelial–fibroblast interactions are believed to contribute to subepithelial fibrosis [65,66]. Since airway remodeling is a marker of many respiratory diseases, a model’s suitability for disease modeling is increased with the incorporation of mesenchymal cells and a suitable ECM.

Co-culturing epithelial cells with mesenchymal cells enables the study of complex airway remodeling and inflammatory mechanisms. Humayun et al. presented a platform to investigate epithelial–mesenchymal interaction by co-culturing epithelial cells with smooth muscle cells separated by a suspended hydrogel. In this system, both cell types remain in separate layers on either side of the gel, and it is possible to remove the cell-laden hydrogel for further downstream analysis of gene or protein expression, or to examine possible matrix deposition or remodeling [22]. This lung-on-a-chip device shows progress towards an effective model for investigating airway remodeling, as paracrine signaling between the two cell chambers occurs through a more physiological ECM substitute. However, the 2D planar configuration of smooth muscle cells on the gels surface does not replicate the 3D configuration seen in vivo, therefore the model’s ability to replicate 3D homotypic cell–cell interactions remains limited.

Lung-on-a-chip models containing tri-cultures have been developed to enable the study of more complex interactions. Although these cultures contain the same main cell types (epithelial, fibroblast, endothelial cells) the microfluidic designs differ. These different microfluidic designs result in a difference in the airway features that are recapitulated. Sellgren et al. utilized a similar approach to previous alveoli models where a porous membrane was utilized to separate independent cellular compartments. To replicate the interstitial space that separates the epithelium and endothelium, an additional microchannel was integrated into the design to create three independent, vertically stacked cellular compartments, each separated by a porous membrane. The apical channel was seeded with primary airway epithelial cells, middle channel seeded with lung fibroblasts, and basal channel seeded with microvascular endothelial cells. Once epithelial cells reached confluency the apical channel was filled with air, while the middle and basal channels were subjected to fluid flow [24]. This approach couples multiple microfluidic chambers to construct a more sophisticated tissue–tissue interface and capture cross-talk between various tissues, although this model’s ability to mimic in vivo like cellular configurations and heterotypic cell interactions is enhanced through the replication of physiologically relevant separation distances and the addition of fibroblasts. The 2D nature of the ECM substitute results in the fibroblasts taking on a non-physiological monolayer structure, limiting the ability to capture 3D cell–cell interactions. The group recognizes this limitation as they stated a further improvement of their model would be accomplished through the incorporation of culturing fibroblasts in an extracellular matrix environment (i.e., hydrogel cage).

Barkal et al. took a different approach where they created a tri-culture model of the bronchioles that leveraged the 3D nature of an ECM hydrogel to embed fibroblasts and create cell lined lumen structures directly within the hydrogel. Lumens were seeded with epithelial and endothelial cells to simulate the airway lumen and blood vessels [27]. This approach enables the creation of structures whose geometry and dimensions mimic the in vivo microarchitecture. Embedding fibroblasts into the hydrogel also enables the replication of 3D interactions. Epithelial and endothelial cells grown in luminal monolayers, in comparison to flat cultures like those grown on porous membranes, represents a more physiologically relevant configuration and enhances direct cell–cell interactions [52,53]. The combination of gel polymerization into a stable 3D structure and pipette-compatible ports incorporated into the model created by Barkal et al. enabled each lumen to be independently accessed. This provides the opportunity to selectively expose cell types to various conditions as well as remove/sample material from specific compartments. It is important to note that although Barkal et al. did not incorporate pump-based flow into their model, microfluidic models of other organs containing cell lined lumens within 3D ECM hydrogels have incorporated pump-based flows highlighting the feasibility of capturing physiological flows in these more complex models (Herland et al. 2016). One of the main challenges of models containing cell embedded hydrogels, is that it can be difficult to collect cells embedded within the gels. This typically requires deconstruction of the device to extract the hydrogel, then digestion of the gel. In multicellular, hydrogel-based models such as Barkal et al. [27], the challenge is exacerbated, as the cells lining the lumen either need to be collected prior to digestion or a cell sorting method is needed to sort cell types.

Park and colleagues also created a tri-culture model of the airways; however, their focus was on using 3D cell printing to create a hybrid model containing a self-assembled vascular platform and a conventional Transwell^®^ airway epithelium model. The model used human dermal microvascular endothelial cells and human lung fibroblasts embedded in decellularized ECM to create a blood vessel network, while primary human tracheal epithelial cells were seeded on a Transwell^®^ insert modified to contain a membrane of the same ECM material [28]. As this model relies on tracheal epithelial cells to mimic the bronchiole, the cellular composition of the differentiated epithelium will be more similar to the trachea than the bronchioles. The self-assembled vessel networks provide increased physiological relevance; however, the mixed culture of endothelial cells and fibroblasts results in increased complexity associated with analysis.

In general, co-culture systems are more physiological relevant, as they mimic the complex in vivo microenvironment better than monoculture systems and can be utilized to investigate direct and indirect cell-to-cell interactions that occur between homotypic and heterotypic cell populations. With appropriate cell selection, ALI and shear forces it has been shown that lung-on-a-chip models have the ability to replicate lung barrier function [20,23,26,49,55], lung injury [17,18], inflammation [23,26,49], and immune response to cigarette smoke [23,59] and infection [23,27,48,55].

In the case of inflammation, models can either stimulate cells in the vascular channel with proinflammatory cytokines (e.g., tumor necrosis factor) or expose the epithelium to irritants (e.g., silica nanoparticles) known to trigger inflammation. To mimic biomimetic exposures these irritants ideally are in an aerosolized format to replicate inhalation mechanics.

Although increasing the complexity of lung-on-a-chip models shows promise for improving physiological relevance—through recapitulation of geometries, dimensions, interfaces (tissue–tissue and ALI), cell–cell interactions, and environment cues—the ability to interpret the data can become difficult as the system become multifactorial and isolating specific interactions can be challenging. In addition to the trade-offs between complexity and interpretability various challenges still exist related to increasing the variety of cells incorporated into a specific model. For instance, each type of primary cell prefers its own specific media, therefore it is necessary when designing complex models to find appropriate media compositions that replicate the in vivo microenvironment and enable all cells to display characteristic phenotypes [24]. Mertz et al. has also identified that current lung-on-a-chip technologies do not address cell-to-cell heterogeneity which can limit the effectiveness of the model as a tool for drug discovery and disease modeling [67].

With the relationships that exist between complexity and predictability, as well as the tradeoffs between complexity and interpretability it is essential to consider how much complexity is needed to investigate the experimental questions of interest. To gain more insight into the interstitial related mechanisms and responses of the lung, both in healthy and disease state, lung-on-a-chip devices need to incorporate interstitial cells (i.e., fibroblasts) and appropriate ECM to capture the 3D in vivo interfaces and interactions.

Table 1 summarizes the cellular components utilized in current lung-on-a-chip devices as well as the in vitro interfaces reproduced.

## 3. Designing ECM Substitutes On-Chip

In addition to mimicking the interactions experienced between cells, further considerations must be made when creating a biomimetic lung model as the interactions between cells, and their surrounding ECM are crucial for respiratory function. It has become increasingly evident that greater attention to incorporating an appropriate ECM into in vitro models is needed to better replicate the native lung tissue [68]. This idea is also reflected in lung-on-a-chip models, where these devices are progressively incorporating more representative ECM substitutes [22,27,28,51,69]. This is of particular importance for lung-on-a-chip models used for studying chronic lung diseases, such as idiopathic pulmonary fibrosis (IPF), asthma, and chronic obstructive pulmonary disease (COPD), where changes to the airway structure and ECM occur [70].

Different microenvironments exist within the lungs, where changes in the composition and microarchitecture of the cells and matrix reflect the specific functions of the localized tissue. Cell behavior, including migration, proliferation, differentiation, protein expression, and gene regulation, can be altered through modifying environmental factors such as ECM composition, structure, and mechanics [71,72]. Conversely, cells can modify their microenvironment through the processes of synthesis, degradation, and matrix remodeling. The interactions between cells and their microenvironment help regulate and maintain homeostasis, while an imbalance in these interactions can indicate and contribute to pathologies seen in chronic pulmonary diseases, such as COPD and IPF [73,74]. For example, increases in collagen deposition is seen in IPF as a result of fibrotic remodeling [75] and elastin destruction is seen in COPD [75,76]. Due to the simplified ECM substitutes used in lung-on-a-chip models, distinct and disease-specific hallmarks seen in chronic pulmonary diseases, such as airway remodeling, still remain difficult to replicate, emphasizing the need for better ECM substitutes [77].

The seminal lung-on-a-chip devices [26,50] comprised an ECM modelled by a thin membrane. As lung-on-a-chip models have advanced, there has been a trend to use three-dimensional hydrogels in place of a membrane to represent the ECM more accurately, as seen by recent models [22,25,28,48]. By classifying lung-on-a-chip devices based on the dimensionality of their ECM, we highlight characteristics of the lung ECM including composition, function, and stiffness that have influenced the progression of ECM substitutes in lung-on-a-chip devices.

### 3.1. Lung ECM

Recreating the ECM in vitro is challenging due to its heterogeneous nature as a dynamic network. As ECM changes with the lung architecture, its representation in a single form, typically as a thin membrane, does not capture the in vivo counterpart. In order to model the ECM, knowledge of the native lung ECM should be considered, where factors such as structure, composition, and mechanics can guide the fabrication or use of the ECM substitute. The ECM substitute should reflect the investigation and the area of focus within the lungs. In relation to investigation, desired endpoints can limit the potential options compatible with current methods. It is also important to note, that as more complex ECM substitutes are incorporated, lung-on-a-chip devices are able to capture more realistic spatial organization of interstitial cells, as well as 3D interactions (cell–cell and cell–ECM). However, the potential compatible readouts and interpretability of the data may be limited.

#### 3.1.1. ECM Structure

In the lung the ECM is the scaffold of the alveolar wall, which consists of the epithelial and endothelial layers, their basement membranes, and the interstitial space between alveolar epithelium and the capillary endothelium [78]. The ECM can be divided into two general forms, either creating the basement membrane or establishing the interstitial matrix [41]. The basement membrane consists of dense layers of glycoproteins, and it lies beneath the epithelial and endothelial layers, where it provides support [79]. The basement membrane connects the rest of the ECM with the cell layer and protects the cells from mechanical stresses [80]. Its major components include collagen IV, laminins, nidogen, and perlecan [81]. In contrast, the interstitial matrix resembles a loose 3D mesh consisting of fibrillar collagen (mainly I and III) and elastin as the major ECM proteins. This matrix provides tensile strength and elasticity to the tissue, and glycoproteins and proteoglycans within the interstitial matrix also resist compressive forces [82]. The interstitial matrix maintains the structure and biomechanical properties of the lungs, interconnecting structural cells. These interstitial cells, consisting of fibroblasts and mesenchymal cells (MSC), play an important part in remodeling and repairing the ECM during growth and after injuries [79,83]. The basement membrane and the interstitial matrix are distinct in structure and composition, therefore the substitutes used to replicate them in vitro should be as well.

#### 3.1.2. ECM Composition, Biomechanics, and Stiffness

The ECM is composed of proteins (including glycoproteins and proteoglycans), polysaccharides, growth factors, and matrix associated molecules, allowing for its multifaceted role [84]. Major proteins, including elastin and collagen I and III, form a mesh of intertwined fibers, which provide the lungs with its characteristic viscoelastic properties [79,85]. Fibrillar collagens provide tensile strength to the lung tissue but have low elasticity, while elastin enables elastic recoil within the tissue [83,86,87]. Soluble proteins including proteoglycans and glycosaminoglycans (GAGs) also contribute to the viscoelastic nature of the lung by forming hydrogels [88]. Viscosity and elasticity are both essential properties of the lung tissue and are required to sustain the mechanical forces of breathing. To describe the stiffness of a tissue or material, the Young’s or elastic modulus, E, is used. It is a measure of the proportional deformation of a tissue or material in response to an applied load. The normal lung parenchyma has an elastic modulus (or stiffness) ranging between 0.44 to 7.5 kPa, which is dependent on the region measured [89]. Environmental stiffness is an important factor as it can affect cells in various ways, including immediate changes in their shape or gene expression [90]. Integrins found in the surrounding environment allow for cell attachment, whereby glycoproteins including fibronectin, collagens, and fibrinogen use integrin receptors to mediate cell adhesion to the ECM [79,82,91]. Through these attachments, cells can sense their environment, acquiring biomechanical and topographical information. Conversely, cells can be controlled through the cell–ECM adhesions, affecting their migration, proliferation, differentiation, in addition to morphology [90,92]. The connection made between the cell–ECM adhesions and cell cytoskeleton allows for feedback mechanisms between the contractile forces, applied by the cell, to maintain equilibrium with the tensile strength of the matrix. Changes in the structure and organization of the cytoskeleton occur through these mechano-sensitive pathways, although particular mechanisms and elements in some feedback connections remain unknown [79].

Although certain mechanisms and elements are unknown, it is known that in the pathophysiology of fibrotic diseases and cancer these mechano-sensitive pathways are important, where dynamic changes in ECM composition and mechanical properties occur with disease progression [93,94,95]. A significant increase in ECM stiffness is seen due to pathologically increased matrix deposition, covalent cross-linking, and ECM remodeling [96]. It is known that ECM stiffness influences cell behavior, including fibroblast spreading, contractility, and differentiation [97]. In chronic pulmonary diseases such as IPF, hallmarks such as fibrosis occur, which cause alterations to tissue composition and lung stiffness [77]. Although it is known that stiffness influences cell behavior, ECM substitutes can be composed of materials that are magnitudes higher in stiffness than native tissues. For comparison, the average measured modulus of human fibrotic lung is 17 kPa [89], while the plastic and glass used in standard tissue culture plates have elastic moduli ranging between 2–4 GPa [98].

### 3.2. Classification by ECM Dimensionality

**Figure 4:**
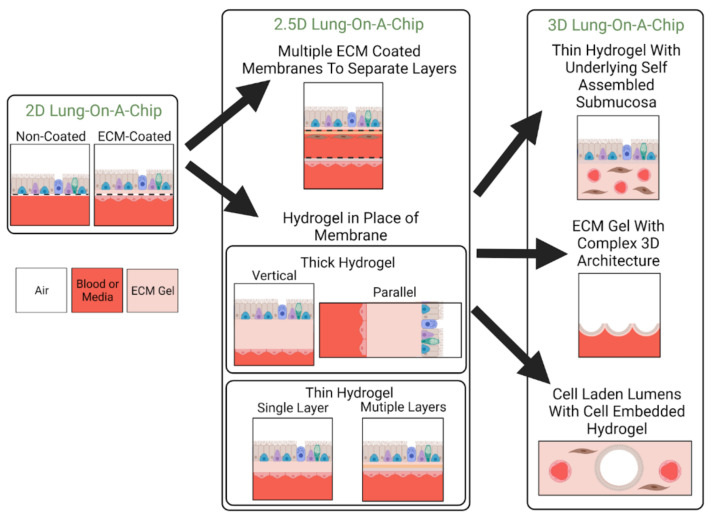
Classification of Lung-On-A-Chip Device Based on ECM Dimensionality. Lung-on-a-chip devices can be made to incorporate ECM substitutes of varying dimensionality. The selected ECM substitute dictates the 3D organization of cellular components as well as how close the model can replicate the native ECM. Here, 2D, 2.5D, and 3D refer to the dimensionality of the incorporated ECM substitute. Devices containing 2D ECM substitutes contain porous membranes that are either left uncoated or are coated with ECM proteins. 2.5D lung-on-a-chip devices either use multiple ECM coated membranes to achieve multilayer stratification or incorporate a hydrogel in place of the membrane to replicate the structure and composition of its native counterpart. Hydrogels can be thin or thick and composed of single layers or multiple layers of ECM material. 3D lung-on-a-chip devices achieve structures more similar to the in vivo setting by incorporating cellular components into the bulk of a hydrogel to obtain 3D cellular organization or creating complex 3D architecture for epithelium. These structures can include alveoli-like pockets or cell-lined lumens.

In this review, we will classify lung-on-a-chip devices based on the dimensionality of their ECM (see Table 2), and define how the terms “2D”, “2.5D”, and “3D” models are used here to discuss the evolution of ECM substitutes in lung-on-a-chip devices for disease modeling. 2D models are defined as devices consisting of a synthetic membrane that is either non-coated or ECM-coated. The main functions of the ECM in 2D models are to: act as a separation barrier between the two compartments, provide structural support for cell adhesion and growth, and allow nutrient or oxygen transport. Additionally, 2D lung-on-a-chip devices are able to cultivate co-cultures, allowing for cell–cell interactions. Depending on the area of focus, 2D lung-on-a-chip models can also have additional functions, such as models replicating the alveoli can contain integrated mechanisms that mimic expansion and contraction emotions experienced during inspiration/exhalation [18,50,56,99]. The 2.5D models generally have a film that is fabricated from ECM constituents and replaces the synthetic membrane used in 2D models. However, as these models use thin films, stromal cells are cultured on the surface in monolayer format rather than embedded within as they reside in vivo limiting their representation of interstitial ECM. Lastly, three-dimensional ECM models incorporate a 3D hydrogel, allowing for cell encapsulation to better recapitulate the interstitial matrix. A visual representation of these classifications is found in Figure 4.

#### 3.2.1. 2D Models: Non-ECM Coated

2D lung-on-chip devices are frequently seen with similar architecture where two channels are separated by a membrane, and dynamic models allow for controllability of at least one flow channel to mimic vascular blood flow [19,100,101]. However, in general, the earlier 2D models incorporated a non-treated membrane as an ECM substitute [19,101]. As such, these models are conducive to planar cell cultures, the simplest to fabricate, and most resemblant of traditional, static Transwell^®^ insert models. Based on Transwell^®^ models, the porous membranes used in these microfluidic devices have been fabricated from the synthetic material polyester (also sometimes referred to as polyethylene terephthalate, PET) [19,101]. Polymer membranes, including PET and polycarbonate (PC), can require fewer steps to integrate into a microfluidic device as they are commercially available in a variety of pore sizes [102]. Both polymers have similar properties as they are transparent and have modulus values in the range of 2–3 GPa [103], which is in the range of tissue culture plastic or glass [98], although it has been reported that, when wet, PET has a measured modulus of 180 MPa [69]. However, this value is still orders of magnitude greater than the lung ECM in vivo, indicating that these membranes are very stiff and rigid. Their stiffness is not much of an improvement when compared to the stiffness of standard tissue culture plates but were used in the earlier lung-on-a-chip models as a starting material.

As these were earlier models, greater emphasis was placed on mechanical functionality and replicating the appropriate cell physiology in the devices. This is reflected in the material choice, based on Transwell^®^ inserts, their limited functionality as a dynamic component, and their limited similarity in biological composition. The membranes used in non-coated 2D models act mainly as a separation mechanism, allowing cells to be separated and organized into an in vivo-like spatial configuration and receive nutrients in a physiological manner. The main feature of incorporating a non-coated membrane as the ECM substitute is its ability to incorporate mechanical actuation into the lung-on-a-chip device itself, allowing for flow in and out of the system. As such, applications of these microfluidic devices focused on utilizing flow to study the effects of air exposure [19] on cell viability and cell layer integrity, as well as studying mechanical stresses experienced by epithelial cells from mechanical injury by liquid plugs [101].

Typically, the 2D lung-on-a-chip models have cultivated only one cell type, often utilizing epithelial cells to imitate the alveolar epithelium. Improved cell adhesion can be seen in the later 2D models where the membranes were ECM-coated.

#### 3.2.2. 2D Models: ECM Coated

The majority of lung-on-a-chip devices in literature fall under the later 2D models which use porous, ECM-coated membranes. The incorporation of an ECM coating promotes cell adhesion for easier cultivation of co-cultures and allows for investigations containing cell–cell interactions. A coating can easily be applied to an existing membrane in a lung-on-a-chip device with an injection or pipetting step. The materials used in these later 2D models are also typically made from polymers, including PET, PC, and polytetrafluoroethylene (PTFE). Additionally, another commonly used synthetic material is polydimethylsiloxane (PDMS), which is advantageous for creating flexible membranes [40,50]. However, perforation of PDMS membranes is required for nutrient or oxygen transport which can pose as a fabrication challenge. Additionally, by changing the ratios of PDMS to curing agent, its mechanical properties can be tuned. PDMS modulus values have been measured at low values within the range of human lung tissue, ~4 kPa [104], to stiff modulus values of several MPa [105]. PDMS also has the ability to withstand mechanical forces and have been previously used in lung-on-a-chip devices for mimicking cyclic stretching in respiration. In their investigation, Douville and coworkers used a flexible PDMS membrane to study the solid and fluid mechanics exerted on alveolar epithelial cells. Although PDMS was chosen for its flexibility, the authors also acknowledged that due to limitations in the fabrication process, their membrane was 100 microns thick—two orders of magnitude higher than the physiological alveolar–capillary barrier [40]. PDMS membrane thickness was later reported to be a magnitude less as Huh et al. demonstrated a 10-micron membrane in their seminal model [26]. Although PDMS has successfully been incorporated into lung-on-a-chip devices, there are some limitations to PDMS, including its hydrophobicity and has been reported to absorb small molecules and drugs [106]. Thus, PDMS is often coated, or surface treated. Coatings used on these porous membranes have consisted of ECM proteins, including fibronectin [18,40,50,107,108] and collagen I [23], fibrous proteins found in the ECM. Additionally, various mixtures of ECM constituents have been used including a laminin, fibronectin, and collagen I mix [99], a PureCol (Col I/III) and fibronectin mix [109], and a gelatin and collagen mix [50]. Coatings promote cell attachment as many ECM proteins contain amino acid sequences (i.e., RDG sequences), which serve as strong cell adhesion sites [110]. Each ECM coating requires optimization based on the cell culture, which is often tested on static ALIs prior to coating lung-on-a-chip devices. However, despite optimization testing prior to cell seeding, cell growth and proliferation might not occur in chips [24].

An important distinction between earlier 2D lung-on-a-chip devices (non-coated) and later 2D coated models is the increased functionality of the ECM membrane as models with coated membranes could provide structural support and allow nutrient transport for one or both sides of the membrane. These ECM substitutes also facilitate co-culturing which enables investigation of heterotypic cell–cell interactions. Additionally, new materials with greater elasticity (e.g., PDMS) allow for lung-on-a-chip devices to be used to investigate the biomechanics of respiratory motion and the effect it has on cell populations. These ECM substitutes offer improvement in terms of cell adhesion and incorporate some native ECM components, however, the tunability of mechanical properties such as thickness, composition, and stiffness remain limited. Many 2D models focus on recapitulating epithelial–endothelial interfaces. These 2D lung-on-a-chip devices force an apical-basal polarity, which is desirable for epithelial and endothelial cells. Whereas alveoli models can use a thin, permeable membrane to model the basement membrane in a 2D model, the two-dimensional nature does not recapitulate the stromal environment needed for studying changes in lung architecture, including ECM remodeling and fibrosis.

Progression towards creating a model that can be used to study these processes can be seen in the lung-on-a-chip presented by Sellgren et al. Their model [24] is an exception to the classification system as it is not a 2D nor a 2.5D representation of the ECM. It utilized a design consisting of three vertically stacked compartments to create a triculture (epithelial–fibroblast–endothelial) model, representing the airways. Each of the three compartments were separated by ECM-coated porous membranes, where the middle compartment represented the stromal layer, and was seeded with fibroblasts. Although this design provided a means to replicate in vivo 3D spatial organization and heterotypic cell interaction through paracrine signaling, the ECM was still limited to 2D. Through optimization testing, they selected PTFE (top) and PET (bottom) membranes with different collagen coatings (IV, I, and I/III, respectively) for each of the different channels. Their use of two different ECM-coated synthetic membranes alludes to a limitation in the mechanical tunability of their ECM model, and those of the 2D models.

#### 3.2.3. 2.5D Models

2.5D models attempt to address some of the limitations seen in 2D models where the aim of the improvements is focused on creating better ECMs [51,69]. Thus, these researchers have moved away from using coated porous synthetic membranes as ECM substitutes. Instead, 2.5D models are fabricated from biomaterials found in lung ECM but use the idea of thin films to represent the ECM. As there are few 2.5D models, they vary in form and function. Notably, Zamprogno et al. [51] focused on the alveoli-blood interface, requiring elastic properties that represent the alveolar sac. The authors focused on the ECM composition and physiological geometry. Their model used a gold honeycomb lattice frame, on which a thin layer of collagen I and elastin mixture was deposited. They modelled an array of stretchable alveolar sacs where the thickness and the stiffness of the membrane can be tuned by the ratios of collagen and elastin in the gel mixture. Their model highlighted the fabrication of a lung-on-a-chip device with an ECM fabricated from biomaterials with more physiological geometry that was capable of cyclic motion. The model represented a progression from the “breathable” 2D lung-on-a-chip models, where they improved on some limitations of using coated PDMS membranes to mimic the flexible nature of the ECM.

In another example, Mondrinos et al. [69] also focused on improvements to the ECM composition, but with a different approach that was guided by the architecture that formed the native ECM. The authors replicated the basement membrane, giving careful attention to mimicking composition and architecture, as they recreated the two layers of the basement membrane: the basal lamina and the reticular lamina. They created the ECM by first creating layers of dehydrated 3D hydrogels, which were then rehydrated and cross-linked using transglutaminase. The ECM was formed by crosslinking the layers in a stack, which also allowed them to tune the thickness and the composition of each layer in the ECM. They had looked at different ECM compositions consisting of collagen I with either Matrigel or alginate. They characterized their ECM including its stiffness and reported the lowest value of 429 kPa. The authors successfully co-cultured various cell types on the ECM, including an epithelial–stromal interface (which was previously demonstrated by Sellgren et al.). Interestingly, through the quantification of cell-adhesions on various ECM models (non-coated PE membrane, fibronectin-coated PE membrane, and their ECM membrane), their findings suggest that cells have greater interactions with their in vitro surroundings (ECM substitutes) when it more closely resembles the in vivo environment. Their model demonstrated a more physiological relevant ECM model, particularly of the basement membrane, where promotive effects of ECM composition on intracellular signaling of mediating cell–ECM interactions were observed. However, their lung-on-a-chip model has limitations in the tunability of its mechanical properties as the additive nature of their ECM fabrication process implies adjustments are incremental. Additionally, their hydrogel films produce planar surfaces, and although representative of the basement membrane, the fabrication process may be less conducive to creating the interstitial ECM.

Although Humayun and coworkers’ lung-on-a-chip model did not encapsulate cells, a natural progression towards a 3D ECM environment can be seen [22]. Their model resembled the classic lung-on-a-chip design mimicking the interface between primary airway epithelial cells (ECs) and smooth muscle cells (SMCs), but was separated by an ECM-laden lamina propria. They represented the lamina propria with an ECM hydrogel consisting of varying concentrations of collagen I and Matrigel. The authors chose collagen I for its abundance in the ECM and Matrigel as it consists of proteins found in the basement membrane. The authors used their “high Matrigel mix” to demonstrate the effects of hydrogel composition on the adhesion and proliferation of the coculture. Collagen I was important for cell adhesion while proteins in the Matrigel allowed for sustained adhesion (7 days).

2.5D lung-on-a-chip models indicate the initial steps toward improving biomimetic ECMs, but challenges in recapitulating the in vivo ECM still remain. The tunability of material properties in 2.5D models, such as thickness and stiffness, are still limited, although improvements from 2D models are evident. It can be noted that from the approach of using thin films, 2.5D models do not support 3D integrin adhesion. However, a benefit that 2.5D models have is their relatively easy fabrication methods as mentioned by both research groups [51,69].

#### 3.2.4. 3D Models

As cells in vivo are immersed in a three-dimensional environment, they experience cell–ECM interactions and interactions with different cell types, along with exposure to gradients of growth factors [111]. It is known that in the 3D environment, fibroblasts show different integrin adhesions [94], polarized acini form from bronchial epithelial cells [94,112], and endothelial cells have increased sprouting angiogenesis [113], which is not normally seen in 2D environments. 3D lung-on-a-chip models attempt to replicate the native ECM through using a three-dimensional and dynamic construct, allowing for cell migration, traction, and integrin adhesion, in the different dimensions, thereby promoting further cell–cell and cell–matrix interactions. Similar to 2.5D models, 3D lung-on-a-chip models can also be fabricated from biomaterials found in the in vivo ECM. However, they are not limited to thin layers made from the constituents, and generally use a hydrogel to represent the ECM substitute.

Hydrogels consist of polymeric materials with high water content (>70 wt%) [114] that form networks with varying structures and properties based on their intermolecular or interfibrillar crosslinks [9]. These scaffolds can be made from natural or synthetic materials, or a combination of both, to produce different material properties. However, lung-on-a-chip devices are generally made of biological materials found in the ECM. Although other biomaterials have been used to create hydrogels in other organ-on-chip models [115,116], in 3D lung-on-a-chip devices, a few hydrogel materials have been reported, including collagen type I [27], Matrigel [22], decellularized ECM [28], and gelatin methacryloyl (GelMA) [25]. Collagen is one of the most ubiquitously used hydrogels, as it is the most common ECM constituent in the body [117]. Collagen is biocompatible, naturally promotes cell adhesion, and sustains many physiological cell functions, making it a popular option for high cell viability, with controlled proliferation or differentiation [116]. Cell proliferation can be controlled by tuning the stiffness of collagen hydrogels, using collagen concentrations or gelation temperature. Non-planar can also be formed in collagen hydrogels through crosslinking, which can be achieved through pH and temperature control [118]. Similar to collagen, Matrigel is another commonly used hydrogel consisting mainly of laminin and collagen (mainly collagen IV), along with some entactin (a glycoprotein found in the basement membrane) [69]. Collagen and Matrigel both have limitations, including batch-to-batch variability, and cold temperatures are required for handling to avoid premature gelation [116]. Gelatin/Gelatin methacryloyl are both similar to each other, and collagen, as they are composed of fibrillar collagen fragments, but additionally, GelMA has functional acrylate groups attached [119]. The additional functional groups allow GelMA to be photo-crosslinked with UV radiation [118]. Gelatin is inexpensive and can be thermally crosslinked.

Progress towards using more representative ECM materials and ease of fabrication can be seen as Park et al. [28] focused on addressing some limitations of previous lung-on-a-chip models by creating an easier and more reproducible method to incorporate a 3D ECM hydrogel. Instead of creating the ECM composition, the authors sought to mimic the in vivo ECM by using decellularized ECM (dECM). They bio-printed various bio-inks, including lung fibroblasts encapsulated in a tracheal mucosa-derived decellularized ECM (tmdECM) and endothelial cells, to form a vascular platform (VP). The upper portion of the PDMS chip consisted of airway epithelial cells cultivated on a Transwell*^®^* insert coated with the decellularized ECM (dECM) and bonded to the bottom VP using oxygen plasma treatment.

Some 3D lung-on-a-chip models demonstrate the possibility of forming structure within the ECM, advancing beyond planar representations. Barkal and coworkers demonstrated the feasibility of incorporating a triculture model of the human bronchiole with a 3D ECM in a lung-on-a-chip device [27]. The model had a central epithelial-lined lumen representing a bronchiole with supporting parallel endothelial-lined vasculatures, where all three lumens were surrounded by a fibroblast-embedded hydrogel made of collagen and fibrinogen. They used a method of fabricating lumens within hydrogels that is easily upscaled. Using their model, they were able to look at an integrated immune response to fungal spore infection. They showed immune recruitment of white blood cells (polymorphonuclear leukocytes, PMNs) through a 3D collagen matrix to the infected lumens fungal spore infection and were able to measure the inflammatory cytokine response. Although this study looked at acute immune response to microbial infection, the lung-on-a-chip device can be used to study other pulmonary pathologies, as it incorporates relevant cell types and a 3D ECM. The microfluidic device is also capable of monitoring changes in ECM architecture through microscopy-based techniques, possibly allowing for studies involving airway remodeling and fibrosis. However, some limitations to the model would include its inability to resample and its lack of fluid flow through the system.

Another recent advancement in developing more representative ECM substitutes is demonstrated by Huang et at. [25]. Their model demonstrated the use of 3D porous gelMA hydrogel to recapitulate the architecture of alveolar sacs rather than the traditional planar model, the current design of many lung-on-a-chip devices. Their device consisted of a main inverse opal structure with sac-like pores and interconnecting windows between the sacs which was bonded to a compartmentalized PDMS chip, allowing for breathing motions. Their model showed similar stiffness to normal human lungs, ~6.23 kPa, and the average size of the alveoli expanded ~8%, which is within physiological range (5–15%) [26]. Additionally, their 3D design allowed for ~7050 alveoli in an 8 × 10 × 3 mm^3^ space, which is also physiologically relevant. They also were able to show that their inverse opal structure, in comparison to planar models (2D PDMS and 2D GelMA), was able to better maintain the functions of primary human alveolar epithelial cells that was more resemblant to the cells in vivo. This reinforces the idea that reconstituting the 3D structures and microenvironments is crucial to our studies of their functions and for modeling pathophysiology in vitro. The study demonstrated the capability of recapitulating the alveolar sac in a more physiological way, that is both architecturally relevant and capable of reproducing important functional features including the microarchitecture, the ECM, the ALI, and mechanical breathing.

## 4. Leveraging Airway On-Chip Technology for Inhalation Assays

When we think of our lungs, we think of breathing—that is, after all, one of the fundamental functions of this organ: to capture oxygen from the air for dispatch through our circulatory system. However, inhaled substances including medications, or toxicants from environmental sources such as cigarette smoke or diesel exhaust, target the epithelial cells that line the airways. To explore how these inhaled substances affect lung function and disease progression, in vitro models should aim to accurately capture the features of the epithelium ALI. Over the past decades, significant advances have been made to accomplish recapitulation of exposure mechanisms and biological responses. The following section of this review examines prior in vitro work, with an emphasis on tobacco cigarettes (a chief causative agent for COPD) and its recently popularized alternative, electronic cigarettes.

Tobacco cigarettes, despite seeing a significant and continued decline in prevalence—especially in industrialized countries—remain one of the leading causes of preventable death worldwide [120,121]. Coinciding with the encouraging decline in cigarette smoking, however, we see a striking increase in the prevalence and use of tobacco alternatives, most prominently electronic cigarettes (‘e-cigs’, ‘vapes’)—especially among adolescents and young adults [122,123,124]. While the former has been widely studied in humans, animals, as well as in-vitro, the relatively recent invention (2003) and rapid adoption of the latter has precluded a comparable-level of study [125,126]. Data regarding long-term chronic exposure in humans is especially limited with electronic cigarettes; this is problematic as it is well established that development of smoking-related disease and pathology occurs through complex and multifaceted mechanisms in response to repeated exposure over a period of years, if not decades [127,128]. Data of this nature is fundamentally difficult to obtain in-vitro; a single in-vitro model cannot be maintained for the durations required to interrogate truly chronic exposure. Instead, such a study would necessitate populations who are regularly exposed to the aerosol of interest (e.g., long-term smokers); the phenotypes of such populations, however, are likely to vary from unexposed subjects (e.g., non-smokers) as well as within the population [129]. The consequences of chronic smoke exposure arise from the compounding of numerous individual acute assaults over a period of time; determining what these consequences may be is imperative and requires longitudinal human studies. Isolating the specific mechanisms that underlie these consequences, however, are where in-vitro studies involving acute exposures are well-positioned to offer insights. In vitro models of the lung are a prime example of these methods.

**Figure 5:**
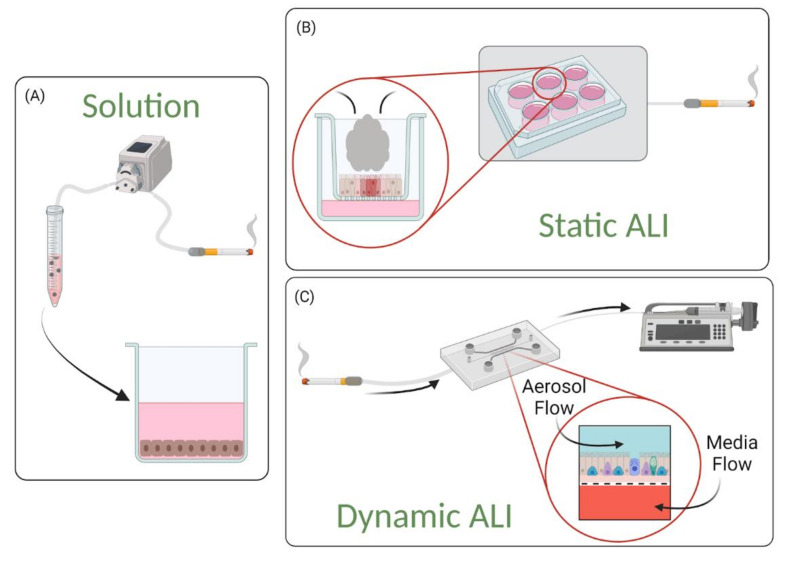
Schematic Description of Exposure Methods. (**A**) In solution-based exposures, a liquid extract is prepared by bubbling the gas through a buffer or cell culture medium. (**B**) In static ALI exposures, the smoke or aerosol is delivered in a single dose to cells cultured in a monolayer at the air–liquid interface (ALI). (**C**) In dynamic ALI exposures, the smoke or aerosol is delivered in a flow profile that mimics the inhalation dynamics.

In general, in vitro exposure of cells to an aerosol of interest is accomplished in three main ways: in solution (where, for example, cigarette smoke is bubbled through culture medium to create a ‘cigarette smoke extract’), as aerosol at a static ALI, or as aerosols at a dynamic ALI (facilitated largely by the organ-on-chip platform) (each illustrated in Figure 5). The first—and prior to the widespread availability of the more recent ALI-based models, the only—method benefits from a high level of congruence with traditional and well-established in vitro culture methods. The principal concern associated with exposing submerged cultures to solutions of an aerosol is the degree to which it diverges from the in-vivo exposure condition—the latter of which we are ultimately concerned with. When an aerosol is inhaled, particulates are subjected to a host of fluid dynamic phenomena which govern their deposition along the airway; these phenomena, furthermore, play a determining role in the phenotypes expressed by the epithelial cells housed in these in vitro models. It has been shown that cells grown at an ALI in vitro better mimic the transcriptional profile of in-vivo epithelia than their submerged counterparts [46]. How well results obtained from solution-based aerosol exposures can be generalized to human health, therefore, is limited.

Nevertheless, even in vivo, cells are exposed not to whole unadulterated smoke, but to a mixture that evolves as it progresses through our respiratory pathway—including extracts in biological fluid [130]. In fact, He et al., exposed mice either to whole cigarette smoke or to cigarette smoke extract through intraperitoneal injection and found no significant differences across the various parameters tested (including lung function, inflammatory cell count, and chemokine release) [131]. In addition to the culture methods, the origins of the cells used in these models can play a significant role; primary cells have the ability to differentiate into in-vivo like airway tissues containing, for example ciliated as well as mucous secreting cells, basal cells, and club cells—immortalized/transformed or cancer cell lines often lack this ability [132]. Furthermore, respiration subjects the airway epithelium to wall shear stress; air flowing across the cells lining our airways during inspiration and expiration creates mechanical forces that have been demonstrated to further regulate cell behavior—for example, modulation of barrier function, mucous production, and ciliary beating alignment [58,133,134]. The choice of in vitro model one makes, therefore, will involve balancing its complexity with the range of physiological phenomena a researcher wishes to recapitulate as accurately as possible. For example, investigating the impacts of electronic cigarette aerosol on ciliary function might require a dynamic ALI, while solution-based exposure may be appropriate for a first-pass comparison in cytotoxicity between cigarette smoke and electronic cigarette aerosol.

### 4.1. Solution-Based Exposures

A number of factors significantly influence the composition of liquid solutions made from aerosol. For example, ‘smoke’—whether from cigarettes or burning wood—is a combination of particulates as well as volatile gases. The post-processing of solutions, therefore, such as filtration to achieve sterility, or lag-time between preparation and use, will affect the quantity of these species remaining in solution. The multifactorial aspect of solution generation results in a difficulty with standardizing protocols. To overcome this challenge, exhaustive detail regarding solution preparation is advisable and can be employed in conjunction with metrics such as optical density, mass spectroscopy, or chromatography (gas and liquid) [135,136,137]. Methods used to generate cigarette smoke extract can be found in Table 3, while eCVE methods can be found in Table 4.

Cigarette Smoke Extract (CSE), a solution prepared by bubbling smoke from ignited tobacco cigarettes through a buffer or cell culture medium, has been widely employed to assess effects and explore associated mechanisms on multiple cell types. Specific preparation protocols can vary notably from group to group; the number of cigarettes bubbled through a volume of solvent—as well as the solvent itself—is not subject to any widely adopted standard. Standardized cigarettes do exist and are produced by the Kentucky Tobacco Research and Development Centre under the University of Kentucky. These reference cigarettes are characterized and manufactured to consistent specification (e.g., tar and nicotine content), in contrast with their commercially-available counterparts that are subject to changing compositions. Due to the volume of literature available on this subject, as well as prior reviews [129,138], this subsection will highlight studies of particular relevance, impact, and interest.

Hoshino et al. exposed A549 cells (a human–alveolar–epithelium-derived cell line) to CSE and found apoptosis induced at concentrations up to 5% and necrosis at 10% and beyond; they found that co-incubation of CSE with oxidant and aldehyde scavengers inhibited apoptosis [135]. Underscoring the sensitivity of aerosol solutions, Hoshino et al. also found that exposing their CSE to open air for 24 or more hours completely attenuated any cytotoxic effects [135]. Carnevali et al. similarly probed a human lung fibroblast cell line (HFL-1) and found CSE dose-dependently induces apoptosis as well as oxidative stress and DNA fragmentation. Co-incubation with the same ROS scavenger employed by Hoshino et al. (n-acetylcysteine) also attenuated these effects [139]. These findings indicate that oxidative-stress-mediated apoptosis may be a significant driver of cigarette-induced injury to both epithelial and mesenchymal components of airway tissues. In 2012, Heijink et al. exposed a human–bronchial–epithelium-derived cell line (16 HBEo-) to CSE and found a transient but immediate reduction in monolayer low-frequency electrical resistance, indicating a loss in barrier function—an important marker of epithelium integrity and health; this effect was attenuated significantly through inhibition of the epidermal growth factor receptor [140].

In addition to cell death, oxidative stress, and barrier function, inflammatory mediators have also been an endpoint of interest, as chronic airway inflammation is a hallmark of COPD—a leading cause of death worldwide, for which smoking is the chief cause [141,142,143]. In vivo, induced sputum of COPD patients contains higher levels of neutrophils as well as their chemoattractant, interleukin-8 (IL-8) [144]. In vitro, CSE has been shown to increase expression and secretion of IL-8 in human bronchial epithelial cells, both primary in origin as well as the tumor-derived NCI-H292 cell line [145,146]. Witherden et al., however, found CSE inhibited IL-8 transcription and release in primary human alveolar epithelial cells; notably, Witherden et al. used a higher stock concentration of CSE, though it was diluted to non-cytotoxic levels (as confirmed via trypan blue exclusion assay) [147]. Kode et al. did find a dose-dependent induction of IL-8 release with exposure to CSE in primary human small airway epithelial cells but failed to replicate this relationship in multiple transformed human alveolar epithelial cell lines—finding insignificant differences in IL-8 release between treatment and control across a panel of lines (A549, H1299, H441) [148]. A potential factor in differing chemokine results may be related to the source of cell within the lung; Thompson et al. found that the neutrophil concentration in bronchoalveolar lavage extracted during bronchoscopy was higher in aliquots collected earlier, termed ‘bronchial’ samples, in the procedure than those collected later (i.e., deeper into the lungs), termed ‘distal’ samples [149]. Osei et al. probed inflammatory mediator release in both epithelial (primary human airway epithelial cells and the 16 HBEo- human bronchial cell line) as well as mesenchymal (primary human fibroblasts and MRC-5 fetal lung fibroblasts) airway tissues; CSE was found to increase the transcription and release of IL-1-alpha in primary epithelial cells and 16 HBEo-, respectively [150]. Exploring cross-talk between the cell-types, incubation of MRC-5 fibroblasts in conditioned, CSE-treated medium from the 16 HBEo-exposure resulted in significantly higher IL-8 release by fibroblasts [150].

In summary, cytotoxicity and apoptosis as well as necrosis, oxidative stress, and reduced barrier integrity have been consistently exhibited by multiple cell types derived from human lungs in response to cigarette smoke solution in vitro, while the literature on cytokine release has been mixed. Multiple cell types found in human lungs are susceptible to CSE-induced effects, and interactions between these cell-types play a complementary role.

Despite the relatively recent advent and popularization of electronic cigarettes, electronic cigarette vapor extract has been the subject of numerous studies. Similar to cigarette smoke extract, a number of considerations surround the generation of aqueous electronic-cigarette-vapor-solution. An equivalent standard such as the Kentucky Reference Cigarette does not exist for electronic cigarettes, nor is there a standard e-liquid. There is a myriad of permutations that exist for the creation and use of electronic cigarette aerosol; the e-cigs themselves vary in type (e.g., refillable vs. disposable pod) and electrical power, while the liquids (consisting of varying concentrations of nicotine dissolved generally in vegetable glycerin or polyethylene glycol) being heated and atomized can contain a variety of flavoring compounds. Many of the endpoints investigated mirror those of CSE; viz. Cytotoxicity, oxidative stress, and inflammatory mediator modulation, and most in vitro assessments have been done on submerged cultures in well-plates using liquid aerosol solutions. Studies involving application of e-liquids to cell cultures have also been completed, though it has been shown that the heating and aerosolization process produces additional compounds and changes the composition of the resulting analyte—as does the voltage at which aerosolization occurs [151,152]. Within the past five years, multiple comprehensive reviews have been written, encompassing in vitro as well as in vivo studies, epidemiological work, marketing tactics, and prevalence, as well as usage statistics [132,153,154,155].

Human airway epithelial cells, both primary as well as multiple cell lines, mesenchymal cells, endothelial cells, and inflammatory cells have been exposed to eCVE. The methods of eCVE preparation can vary in volumes of aerosol or mass of liquid aerosolized in a given volume of solvent; some groups utilize continuous flow (e.g., peristaltic pump driven), while others mimic established puff regimes (e.g., the ISO standard dictating 35 mL puff over 2 s with an inter-puff duration of 28 s) [156,157]. Characterization of extracts was also commonly, though not universally, employed and involved similar techniques to those employed with CSE (gas chromatography–mass spectrometry, optical density). Instead of dissolving aerosol into culture medium prior to exposure, Cervellati et al. delivered whole aerosol to an incubator occupied by submerged culture in lidless well-plates, allowing dissolution into medium to occur during the experiment [158]. Higham et al. employed an ALI to establish culture but exposed cells to a liquid electronic cigarette vapor solution [136]. Results with respect to cytotoxicity, oxidative stress, barrier function, and cytokine release were mixed, with some groups finding correlations with flavor and nicotine content [156,158,159,160]. In all cases, effects were lower than those seen with exposure to CSE.

### 4.2. Static Air–Liquid Interface (ALI) Exposures

Culture of airway epithelial cells at an ALI generally involves the use of permeable membrane supports (e.g., Transwell^®^ inserts). These supports allow for the seeding of a cell suspension, which, after cellular adhesion, can be raised to a level corresponding to the media interface. The porous membrane facilitates the creation of a polarized epithelium and allows for nutrient and biomolecule exchange. For details regarding culture protocol, please see the example presented by Karp et al. [164]. ALI culture systems allow for whole aerosol to be delivered to airway epithelia—thereby exposing cultures in a more in-vivo-like context without sacrificing the non-water-soluble components lost with dissolving aerosol in culture medium.

A consideration for air–liquid interface culture, in general as well as during exposure studies, is mucus production and mucociliary clearance. A hallmark of ALI differentiation into in-vivo-like airway epithelial tissue is the presence of ciliated as well as mucus producing (goblet) cells [164,165]. Static ALIs, which generally leverage Transwell*^®^* insert technology, comprise a cell-layer encircled by a tissue culture dish; as such, cilia cannot clear mucus as they would in-vivo, which results in a layer of mucus overlaying the culture [164]. The presence and properties of this layer play a role in the absorption of molecules in aerosols or drug formulations delivered to these cultures [166]. Rates of mucus production in-vitro depend on cell type and the presence of regulatory factors (e.g., ATP and UTP), and studies that have explored mucus production rates in primary cells as well as cell lines often use indirect, semi-qualitative measures such as relative optical intensities generated from Alcian Blue staining or mRNA expression levels [167,168,169,170]. Abdullah et al., however, provided an absolute quantitative measure of baseline mucus release of approximately 200 ng per hour per culture (12-well Transwell*^®^* inserts) from normal primary human bronchial epithelial (HBE) cells by washing cultures at regular time intervals and assaying aliquots for dissolved mucin [171]. Presence of mucus can interfere with assessment of certain assays and endpoints (such as TEER measurement); the most common method of clearing mucus in static ALI cultures involves washing the apical compartment with phosphate-buffered-saline (PBS) or culture medium [172]. Submerging the apical compartment in a mucus solvent, however, undermines the air–liquid interface; while this may be for a short time, changes in cell phenotype may be induced, confounding exposure-related responses [173]. Some groups have reported regular washing of apical compartments for mucous clearing at weekly intervals during culture maintenance phases, as well as days prior to exposure to minimize confounding [172,173]. Longer-term exposure studies involving cigarette smoke or electronic cigarette aerosol involve intermittent exposures conducted over time intervals not longer than a couple of weeks; such intervals may not be long enough for mucus accumulation to become a significant issue, and regular washing protocols are not reported [174,175,176].

Cigarette Smoke has been widely employed in ALI culture systems. A comprehensive review focused on this topic was published in 2016 by Li [177]. That review summarized research conducted on a variety of airway epithelial cell lines as well as primary human airway epithelial, alongside fibroblasts alone or in coculture [177,178]. Common endpoints investigated through these studies include cytotoxicity, oxidative stress, and inflammation. As found with extract studies, cytotoxicity and oxidative stress exhibited a dose-response relationship with whole cigarette exposure; IL-8 and IL-8 expression was also increased with exposure to whole cigarette smoke in primary human bronchial epithelial cells and the NCI-H292 and MM-39 cell lines [179,180]. Ciliary function was also probed and found to be adversely affected, with cilia counts and beat frequency reducing [176,178]. Conducting whole smoke aerosol exposures requires adaptations to conventional cell–culture approaches. The majority of studies utilized either the commercially available CULTEX^®^ or VITROCELL^®^ exposure systems, which are purpose-built to deliver aerosol to Transwell*^®^* cultures; publications characterizing these systems with dedicated explanations regarding operation and use are provided here [181,182,183,184]. Beisswenger et al. employed a custom system, whereby cigarette smoke was pulled via pump through an exposure chamber containing Transwell*^®^* cultures [180]. Interestingly, St-Laurent et al. found a different cytokine release profile between primary rat bronchial epithelial cells exposed to CSE under submerged culture and to whole smoke at ALI [185].

Effects of exposure to whole electronic cigarette aerosol have also been studied under ALI. Mechanisms of exposure utilized human airway epithelial cell lines as well as primary cells cultured on Transwell*^®^* supports, with aerosol delivery accomplished via CULTEX^®^, VITROCELL^®^, or a custom chamber/pump configuration. Parameters investigated included cytotoxicity, oxidative stress, barrier integrity, cytokine release, and ciliary beat frequency. Results regarding cytotoxicity and oxidative stress were mixed, though release of IL-6 and IL-8 was commonly found. Iskandar et al. found insignificant effects regarding ciliary beat frequency with ECA exposure [186]. A summary of exposure methods, cells used, and experimental results for static ALI exposures to electronic cigarette aerosol is provided in Table 5.

### 4.3. Dynamic Air–Liquid Interface (ALI) Exposures

Representing the most in vivo-like exposure method, dynamic ALIs are an emerging and relatively recent tool. A dynamic ALI exposure system can also permit study of aerosol deposition and how aerosol size affects deposition outcomes. A dynamic system can be used to study how convection, sedimentation, and Brownian diffusion affect the deposition distribution, and can be used to validate in silico models [194]. Deposition will be of particular interest for disease where there could be airway obstructions or inflammation, as the deposition will differ greatly from that in healthy airway trees. Existing methods to study aerosol deposition in vivo indicate coarse-grained regions of interest [195] and do not give high resolution maps of precise deposition patterns.

The only example of a dynamic exposure to cigarette smoke at the time of writing was conducted by Benam et al. [60]. They employed a PDMS based microfluidic chip, within which a rectangular channel seeded with primary BECs (from COPD patient as well as healthy controls) atop a PET membrane (comparable to those comprising Transwell^®^ supports). This membrane separated the channel into an apical and basal section with the latter perfused with culture medium. Cigarette smoke from Kentucky reference cigarettes was delivered to the apical channel using a syringe-pump that drove bi-directional flow to simulate human inspiration and expiration. They found an upregulation of antioxidant genes through rt-qPCR, in accordance with results well-established throughout static ALI and solution-based exposure studies. Leveraging the real-time visualization of transparent microfluidic systems, video microscopy was employed to assess induced ciliary dysfunction. A change in the beat frequency distribution was found, where increases in the low-frequency range and decreases in the high-frequency range accompanied exposure to cigarette smoke. Cytokine release was also assessed; IL-8 secretion was significantly higher following exposure to cigarette smoke, though, interestingly, only in COPD-patient derived cell samples. This model was also applied to investigating electronic cigarette aerosol, where no significant changes were found with either oxidative stress or ciliary beat frequency. Benam et al. recently published a comprehensive protocol detailing design and fabrication of an exposure system compatible with microfluidic airways-on-chip [196]. Li et al. employed a PDMS microfluidic device into which primary BECs from COPD and lung cancer were seeded; the exposure was conducted using liquid CSE, which was found to induce proliferation at low concentrations but apoptosis at high concentrations, generation of reactive oxygen species (indicative of increased oxidative stress) and promoted the epithelial-to-mesenchymal transition—as qualified by modulation of E-cadherin and Vimentin expression [29]. Hou et al. also employed CSE in a microfluidic model; in this case, a co-culture of BEAS-2 B BECs and HUVEC endothelial cells were seeded into the device separated by a porous membrane. Following exposure to CSE, IL-6 levels were elevated, as was expression of TNF-alpha; several apical-junction-related genes were also affected, indicating adverse effects on barrier function [21].

The concern described in the section preceding regarding mucus production is more pronounced in lung-on-a-chip models, as mucus accumulation may lead to obstructions in apical compartments or in downstream tubing. Benam et al. employed a weekly apical compartment rinse with culture medium to counteract this [23]. The exposure protocol they employed was 75 min in duration, with assessment the following day; as such, mucus management was not of concern [60]. Longer-term dynamic exposures on the order of weeks, however, may require intra-exposure management of mucus; this poses a difficult challenge as perfusion of the apical channel with a wash medium to clear mucus may confound responses due to exposure. Perfusion with clean, humidified air between exposure might enable mucociliary clearance out of the lung-on-chip, while regular replacement of downstream tubing and connections may prevent blockages and eliminate the need to perfuse with liquid, preserving an ALI. The ability to remove mucus without the need for intermittent submerged culturing highlights how the microfluidic nature of lung-on-a-chip devices can potentially capture aspects of airway biology unattainable in other standard methods.

## 5. Readouts from Lung-On-A-Chip Models

In addition to the architectural design and cellular make-up of lung-on-a-chip devices, it is also essential to monitor the physiological state of organ-on-chip tissues, and their response to stimuli or compounds in drug screening. Therefore, the methods selected for characterizing the tissue model are just as important as the components utilized to create them. With traditional in vitro models, characterization tends to rely on endpoint-based assays and staining. Organ-on-chip systems, incorporating natural ECM components, multiple lung cells, with dynamic airflow and perfusion systems, are compatible with many traditional methods but can also be integrated with sensors for both real-time and endpoint analyses. The combination of material properties, microfluidics, and compartmentalized design offers the ability to evaluate samples in situ in a real time or continuous manner using various methods.

Most lung-on-a-chip models are constructed from optically transparent materials, such as polydimethylsiloxane (PDMS) or polymethyl methacrylate (PMMA), that provide the opportunity to capture the real time changes seen within the models [197,198]. In terms of airway remodeling and repair, real time analysis measurements would facilitate the investigation of cell–cell interactions including epithelial–mesenchymal interactions, as well as fibroblast–ECM interactions including ECM deposition, degradation, and reorganization. Leveraging the real-time capabilities of microfluidic devices provides the opportunity to gain further insight into the biology and pathophysiology of the lung, especially when investigating underlying mechanisms that cannot be assessed through end-point analysis.

To date, most real time analysis readouts that are compatible with lung-on-a-chip technology require microscopy and/or biochemical analysis on the collected effluent. Consideration of the desired characterization methods and endpoints during the device development or selection process is crucial, as devices may need to incorporate design features that facilitate their compatibility with existing protocols, equipment, and assays. Common features incorporated into lung-on-a-chip devices to enable real time and/or continuous measurement include glass bottoms optimized for fluorescence and confocal microscopy, sampling ports and/or sensors (on-chip and in-line). Alternatively, if a device is considered incompatible with the traditional methods used to assess a desired endpoint, then focus could be placed on creating new characterization methods designed specifically for the organ-on-a-chip technologies. Various emerging methods such as label-less detection have the opportunity to further enhance the real-time capacity of lung-on-a-chip models.

### 5.1. In Situ Characterization and Real Time Analysis

**Figure 6:**
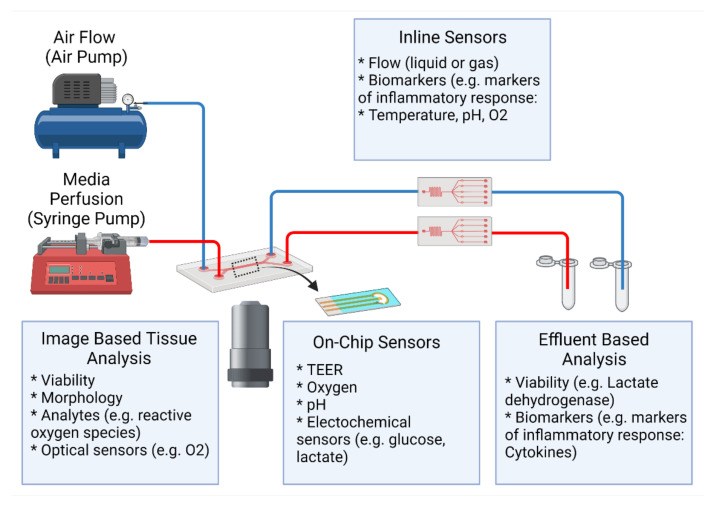
Readouts from Lung-On-A-Chip Devices. Lung-on-a-chip technologies allow time-varying, dynamic inputs to the microtissues, such as flow (both media flows to mimic blood flows as well as gas flows to mimic inhalation) or biochemical stimulation. These platforms can be integrated with technologies to monitor the outputs. Continuous measurements can give information about evolution of the biological response, while snapshot endpoint measurements can also be adapted to the microscale platforms. On-chip sensors (e.g., optical, electrical, electrochemical) are integrated within the organ-on-chip device itself. In-line sensors are external sensors that can be used to monitor the input and/or outputs to the chips.

One of the major advantages of lung-on-a-chip technologies is that it permits real time analysis of lung biology and response. However, it is important to note that the microscale aspect of these devices pose some challenges in relation to traditional techniques that need to be overcome. Challenges include air bubbles compromising image quality, limitations in imaging penetration depth and resolution, and small sampling volumes resulting in low signal-to-noise ratios and limited biological materials. Microfabrication techniques offer ample opportunities for integrating sensors directly on-chip, however in-line sensors can also provide great insight. Figure 6 highlights the types of real time analysis that can be conducted in situ with lung-on-a-chip technologies.

Barrier integrity is an essential in vivo characteristic of the lung that needs to be replicated in lung-on-a-chip models. To characterize barrier formation and function, real time methods using fluorescently labelled paracellular permeability markers or integrated sensors are advantageous over end point methods, relying on immunostaining for junction proteins.

#### 5.1.1. Microscopy and Imaging Capabilities

Imaging techniques are an integral aspect of analyzing lung-on-a-chip technologies. The optical transparency of the materials used to create lung-on-a-chip devices facilitates real time visualization of the structures and architecture of the 3D culture contained within the microfluidic device, as well as enables monitoring of cellular interactions enabling easier in situ characterization [198]. Lung-on-a-chip devices are capable of end-point analysis or live cell imaging, where end-point analysis methods use standard or slightly modified microscopy techniques.

One of the simplest imaging modalities that could be used for live cell imaging of microfluidic devices is widefield fluorescence microscopy (WFM). If the sample is a monolayer, then simple assessments during lung-on-a-chip device culturing, such as cell morphology and confluency, can be checked using this technique. Although WFM is an old technique, its simplicity and low photon dosage can make WFM an attractive choice [199]. However, WFM has limitations in obtaining quantitative assessment. Careful consideration of an experiment’s functional output can result in appropriate data, where WFM can provide a convenient and effective method for analysis. In the study by Benam et al., 2016, high-speed microscopy has been utilized to confirm the presence of cilia beating. Cilia, which are present on the apical surface of the epithelium, beat in unison to push mucus and trapped particles upwards towards the throat. Benam et al. were able to assess cilia beat frequency and confirm that their lung-on-a-chip model replicated frequencies (9–20 Hz) similar to those observed in healthy human airways [23].

With the use of fluorescent probes (i.e., fluorescent dyes, trackers, fluorescent proteins), live cell imaging can be conducted to assess various cellular responses using optical modalities such as confocal microscopy or multiphoton microscopy (MPM). Confocal fluorescence microscopy is a popular analysis method utilized to characterize 3D cell and tissue models [200]. The method can be used to analyze localization and quantity of different cell types or specific proteins within the model. One of the main considerations of confocal microscopy is the choice of fluorescent markers for cell labeling. This either results in the need to transfect cells with fluorescent proteins (FP) or utilize a fluorescent probe. FPs are commonly used for visualizing movement of live cells as a label-less method after initial transfection and have been demonstrated on a lung-on-a-chip device. Huh et al. demonstrated the cellular response of the underlying endothelium to GFP expressing E. coli bacteria that was introduced onto the surface of alveolar epithelium. They captured the transmigration of neutrophils into the upper channel, where the neutrophils then proceeded to engulf E. coli bacteria in minutes [26]. However, some limitations of FPs include the need for cell transfection, which can be particularly difficult with primary cells [201], their large size which can affect cellular activity or localization of the FP [202], and being potentially toxic to cells [203].

In the case where cells are not expressing fluorescent proteins, fluorescent probes such as CellTracker^TM^ (Invitrogen, USA) can be used for live cell imaging. For example, Hou et al. labelled BEAS-2 B and HUVECs with CMFDA (green) and CM-DIL (red), respectively, to facilitate live cell imaging [21]. The long timelines required to achieve successful differentiation of epithelial layers can limit the compatibility of short-term probes such as CellTracker^TM^ as they may require re-staining of samples for longer term monitoring; a similar case arises for Hoechst staining. Some limitations to using confocal fluorescence microscopy for live cell imaging for lung-on-a-chip devices could be the penetration depth, as some models contain tissue layers on the order of several hundred microns thickness, light scattering within those tissues, and the potential autofluorescence from biomaterial scaffold materials, as certain hydrogels have been reported to produce an autofluorescence [204].

Multiphoton microscopy (MPM), including two-photon microscopy (TPM), is also commonly used to study airway remodeling and can be used for live cell imaging. MPM can be used to increase the imaging depth and a variety of fluorophores also fluoresce under multiphoton imaging. However, its use in lung-on-a-chip devices is limited despite its wide use in pulmonary research [205]. MPM enables both fluorescent and second (or third) generation harmonic (SHG) microscopy which would be beneficial when studying airway remodeling on chips. The longer excitation wavelengths of MPM allow for imaging of thicker samples, and less photodamage to samples which is an important consideration when continuously monitoring [206]. Furthermore, as MPM uses longer excitation wavelengths in the red and near-infrared spectrum, there is greater availability in choice of additional probes. Conversely, if fewer probes are desired, MPM can be advantageous when used in conjunction with confocal microscopy, especially for visualizing the ECM. Major proteins, fibrillar collagen and elastin, can emit endogenous signals under these microscopies, allowing for label-free visualization of the ECM structure [207]. For example, leveraging SHG could provide the ability to assess the presence, distribution, and organization of collagen without relying on fluorescent dyes, while two-photon-excitation-fluorescence microscopy can be used to monitor elastin. This technique is commonly used to study the organization of collagen hydrogels [208] and ex vivo samples [209]. MPM has many biomedical applications, including the characterization of airway remodeling in asthma, where assessment of structural changes to lung ECM suggests chronic accumulation of disorganized fibrillar collagen [210].

Another image-based method to consider when fluorescent markers are used is spectral imaging. Some confocal and multiphoton microscopes have the capability of spectral imaging which can be advantageous when many probes are involved. Generally, fluorescence-based imaging is limited to using a few fluorescent markers simultaneously due to spectral overlap of their excitation and emission. However, spectral imaging combines optical and spectroscopy methods to acquire detailed information of the sample and uses algorithms to “unmix” the signals. Although there are two forms, emission-based and excitation-based, emission-based spectral imaging is more relevant as it entails collecting the entire emission spectrum at every pixel, more resemblant of conventional fluorescence-based microscopy [211]. Often, spectral imaging is used with fluorescent probes (where a probe’s emission spectrum is known) and captured using spectral detectors. Essentially, every fluorescent probe has a distinct spectrum that is characteristic to their molecular composition that can be used to identify each probe [212]. Thus, regardless of spatial overlap, by using algorithms such as linear unmixing, the fluorescent markers’ spectrums can be distinguished from each other, as they are unique. This can be advantageous in capturing multiple cell types, as is often seen in lung-on-a-chip devices. Spectral imaging pairs well to capturing dynamic changes as the fluorescent probes can be separated regardless. Cohen et al., 2018, demonstrated spectral imaging of six probes simultaneously at subcellular level [211]. When thought out, spectral imaging can be used to study cell–cell or cell–ECM interactions. However, some limiting factors to using this modality would include the image background and detector noise especially when using thick samples. Additionally, the choice in the spectral resolution should be considered as this can cause weak signals [213].

To leverage these image-based modalities for cell–ECM interactions, considerations of labelling not only the cells, but the microenvironment as well could allow for greater detection capabilities. Recently, Lu and coworkers [214] generated both GFPtpz and mCherry-tagged collagen fusion proteins which were successfully expressed in murine osteoblast-like cells and embryonic fibroblasts. They used live cell imaging to capture the highly dynamic nature of collagen assembly, and were able to show that during assembly, the underlying cell network causes continual stretching and contracting of the fibril networks. Their collagen tagged fusion proteins, along with any of the fluorescence or spectral microscopy, can be effective at label-less detection of cell–ECM interactions. Adapting this to lung-on-a-chip models could allow for long-term studies and continuous monitoring of cell–ECM interactions, such as matrix remodeling for chronic pulmonary diseases.

Furthermore, as lung-on-a-chip devices begin to incorporate 3D hydrogels to mimic the ECM, another consideration in utilizing the microenvironment for dynamic studies could include the use of stimuli-responsive or “smart” hydrogels. These hydrogels can respond to external or internal cues to organize their architecture, typically through swelling and un-swelling, or changes in stiffness [215]. These responses are reversible with cues that include: thermo, pH-based, electro-sensitive, and light responsive hydrogels. Of particular interest are the light sensitive hydrogels, as activation through a light source coupled with imaging techniques can allow for real time monitoring of cellular or multicellular responses to environmental changes. Thus, the considerations for using reversible stimuli-responsive hydrogels could align well with studying matrix remodeling. These advancements have the potential for a myriad of investigations involving chronic lung diseases.

There are many important considerations for live cell imaging with lung-on-a-chip devices, which include but are not limited to: the sample and the experiment, the microfluidic chip design, the optical setup, and equipment. As this is not the focus of the section, lung-on-a-chip device examples will be used to briefly illustrate the considerations.

Example 1. Sample and Experiment: The sample and experiment are two of the most important considerations as it can influence the other consideration factors. Understanding the limitations of a sample and what is required of the experiment is crucial to live-cell imaging choices. For example, in the lung-on-a-chip model from Thacker et al., they used time-lapse imaging to quantify bacterial growth rates of a murine tuberculosis (TB) model which was a direct method in comparison to the indirect quantification of bacterial growth rates seen in mouse models. Additionally, the authors specifically chose murine cells rather than human cells in order to add constitutively-expressing-GFP mice macrophages to the lung-on-a-chip device. In this way, they could clearly identify these cells over many days, which is not possible with short term probes like CellTracker^TM^ and other fluorescent dyes [216].

Example 2. Microfluidic Chip Design: Modifications to either the microfluidic chip design and/or the optical setup is commonly done for imaging microfluidic devices. Similar to other complex *3D* cell cultures, increased complexity can result in samples that are difficult to image. Hence it is essential that the complexity of the model is weighed in contrast to ease of imaging and data interpretation. Constructing models atop a glass coverslip can improve imaging quality by decreasing the penetration depth needed to successfully image the contained cell culture [27].

Example 3. Microfluidic Chip Design: Some Lung-on-a-chip devices utilize parallel channels (Figure 2C,F) in contrast to vertically stacked channels, to aid in imaging [20,27]. However, in parallel format gravitational forces are eliminated and it is important to note the effect these structural simplifications have on cell behavior and response are unknown. In the Barkal et al. lung-on-a-chip model, flow is not used and therefore the design did not consist of needles or tubing, allowing for live cell imaging and their investigation of immune recruitment to microbial infection [27].

Example 4. Optical Setup and Equipment: Additional considerations to optical setup are needed when imaging a lung-on-a-chip device for live cell imaging or long-term monitoring. Standard microscopy equipment such as stage-top incubators are available, but may not be compatible with microfluidic chips that have tubing, or protruding inlet ports, for delivery of the treatments. Custom-made or in-house modifications can be made to the optical setup, allowing for better imaging. For example, Peel and coworkers developed an automated imaging process for their microfluidic device, where higher throughput imaging of multiple chips was possible. They designed and 3D printed an adapter to their microscope stage, consisting of three parts and used an imaging algorithm to optimize the automation of the imaging process. Although the model was a kidney-on-a-chip device, similar or highly relevant considerations could be made when imaging lung-on-a-chip device [217].

#### 5.1.2. Effluent Collection and Supernatant Based Assays

Unlike other 3D cell cultures (i.e., organoids), the microfluidic nature of lung-on-a-chip technologies facilitates in situ analysis of metabolic and biochemical activities of living cells. Incorporation of inlet/outlet ports in combination with sampling techniques enables the continuous collection of supernatants that can then be processed using standard off-chip techniques (i.e., enzyme-linked immunosorbent assay (ELISA)) to investigate the presence of various markers of lung function and response. Cytotoxicity can also be probed by quantifying the release of factors associated with cell death, such as lactate dehydrogenase or adenylate kinase. Cytokine production/secretion has been extensively analyzed on lung-on-a-chip models with the help of ELISA [21,23,27,28,50]. Common inflammatory markers assessed in lung-on-a-chip devices include IL-1β [27], IL-6 [21,23], IL-8 [23,27,50], and TNF-α [21]. The advent of multiplexable ELISA kits can increase the throughput of this process, furthermore, by allowing multiple secreted biomolecules to be quantified in effluent simultaneously. In addition to the upregulation in inflammatory response that these components have, many play a role in fibroblast proliferation, myofibroblast differentiation and collagen production, therefore having the capacity to analyze these components in a continuous manner will provide further opportunity to gain insight into airway remodeling.

Similar to imaging, bubbles can alter cell response and compromise the ability to collect samples that reflect the in vitro environment. In addition to bubbles, the continuous flow that is being passed through the device can complicate analysis. ELISAs are a sensitive immunoassay (typical detection range of 0.01 ng to 0.1 ng) and are therefore compatible with small volumes, however the operation of a microfluidic chip differs from the traditional static in vitro models. In static models, factors are released into a set volume that does not increase over time resulting in a concentrating effect. Generally, in microfluidic devices, the volume collected as effluent continuously increases resulting in a diluting effect. The diluting effect is dictated by the selected flow rate, with faster flow, which correlates with higher shear, resulting in an exacerbation of the effect. The difference in operation between static and dynamic culture emphasizes that in order to collect standardized aliquots of culture supernatants there is the need to be diligent about sampling times as well as factoring in any potential dilution that may result. The incorporation of hydrogels into the models can further impact these types of measurements as cytokines, chemokines and growth factors must diffuse through the ECM layer out of and into the vasculature compartment for collection.

To overcome the limitations associated with small volumes of cellular materials, chips can be pooled together, although this approach overlooks potential discrepancy between samples and involves culturing large numbers of chips. Alternatively, as the size scale of microfluidics is closer to that of high throughput screening, methods commonly used for high throughput screening can be more easily adopted for organ-on-a-chip devices. It is also important to note that the cell culture volumes are small in comparison to the dead volumes of the microfluidics system therefore this needs to be considered in the interpretation of the data [218]. For example, if a chip contains two 20 µL vertically stacked chambers (total culture volume of 40 µL) but requires 25 cm of 0.02″ID tubing at the inlet and outlet ports to connect to a pump and effluent collectors than the dead volume to cell culture volume ratio would be approximately 2.5:1. Nalayanda et al. were able to increase the compatibility of their lung-on-a-chip device with traditional assays such as TEER and surfactant droplet test by incorporating an open system on the apical side to improve ease of access to the cells [19].

Endpoint measurements of interest during exposure studies include cytotoxicity, cytokine and inflammatory mediator release, barrier function, and oxidative stress. Both cytotoxicity as well as cytokine release can be assayed with straightforward adaptations (i.e., collection and transfer of effluent from lung-on-chips into well-plates). Measuring barrier function and oxidative stress, however, pose additional challenges. The former is commonly assessed via measurement of electrical resistance across an epithelial layer, and discussion of its applicability to airway-on-chip devices was discussed above. Diffusion of molecules across an epithelial layer is another measure through which barrier function can be assessed; fluorescein isothiocyanate (FITC)-dextran is commonly employed to this end [219]. Assays of effluent aliquots from an airway-on-chip’s basal channel at a time point following introduction of the FITC into the apical channel provides a mechanism to assess barrier integrity across control and treatment pairings in-situ with minimal modifications to the microfluidic chip [220]. A limitation of this method, however, lies in the liquid phase of reagents (compromising an ALI) as well as the temporal delay required (measurement resolution will depend on the amount of diffusion time allotted, as opposed to real-time electrical sensing of TEER). Challenges associated with permeability-based assay increase in number when lung-on-a-chip devices contain ECM hydrogels, as unlike those separated by a porous membrane, the fluorescent marker must diffuse through the hydrogel into the vasculature channel for collection. Attenuation of fluorescent markers within the ECM may confound measurement of barrier integrity. To overcome this complication, sampling time points should be selected based on the diffusion characteristics of the gel. Oxidative stress assessment involves measurement of intracellular redox balance; this is commonly accomplished by quantifying ratios of oxidized and reduced glutathione (through HPLC, for example) or through the use of probes that fluoresce depending on their redox state (2′,7′-dichlorodihydrofluorescein diacetate) [139,190]. The former method requires cell lysate analysis, while the latter may theoretically be applied in-situ by perfusing the microfluidic channel of interest with a solution of the fluorescent probe (which would also undermine an air–liquid interface).

Both barrier function as well as oxidative stress, however, can both be interrogated through transcriptomics approaches; these have the added benefit of assessing other gene targets. In-situ lysis of an epithelial layer was performed by Benam et al. for qRT-PCR analysis of oxidative-stress-related genes as well as microarray analysis [60]. Barrier-function-related-genes, such as those coding for apical junction proteins, have been used to assess differences in barrier integrity with stimulation [21]. Augmentation of such assessment with direct measurements is ideal, if available, as statistically significant differences evidenced by FITC-dextran or TEER are not always mirrored exactly in junction protein expression [175]. Transcriptomics is a promising application to microfluidic chips; lysis buffers can be injected into cell-containing channels and lysates can be collected as effluents. These effluents can then be used with specific primers for relative and absolute expression levels of a gene-of-interest via qRT-PCR, in conjunction with microarray analysis to interrogate numerous targets simultaneously, or in conjunction with NGS RNA-seq to explore transcriptome-wide expression differences [221,222]. A potential limiting factor with in-situ lysis, whether for transcription analysis or protein qualification (e.g., via Western Blot), is the potentially low cellular-yield extractable from microchannels discussed above. For example, the recommended input for Single-Cell RNA-Seq is 1 million cells (GENEWIZ^®^, Seattle, WA, USA), however the number of cells contained within microfluidic devices can be in the thousands.

#### 5.1.3. Integrated On-Chip Sensors

In addition to its compatibility with microscopy and effluent based assays, lung-on-a-chip technologies provide the opportunity to integrate on-chip microsensors for real-time detection. Sensors prove a way to improve the real-time readout capacity of microfluidic systems. Microsensors, electrochemical or optical in origin, which can detect cells and environmental conditions, have been incorporated into lung-on-a-chip devices to assess barrier function in real time [223,224,225,226].

Transepithelial/endothelial electrical resistance (TEER) is broadly used barrier integrity readout employed in traditional in vitro lung models that rely on culture insert. In vivo, epithelial and endothelial cells form confluent monolayers wherein the cells are connected to each other through intercellular junctions. Tight junctions allow the cell layers to regulate transport between the apical and basolateral sides and maintain homeostasis. For example, infection or inflammation can lead to gaps in the endothelial layer, resulting in increased barrier permeability and eventually to edema [227]. TEER utilizes electrical probes to determine the electrical resistance across epithelial or endothelial layers. In good barriers, the electrical resistance is high, indicating a tight, confluent monolayer. Due to their small volumes and closed nature microfluidic platforms can be incompatible with traditional TEER methods. The small volumes can result in low signal-to-noise ratio, while the closed design can prevent the introduction of electrodes into the various channels. To overcome the challenges associated with using traditional TEER methods, devices can be modified to enable easier access for probes [19] or electrodes can be embedded into the microdevice design to improve signal-to-noise ratios and facilitate real time, non-invasive monitoring of TEER [223,224,225,226]. The incorporation of embedded TEER electrodes also provides the opportunity for quality control of the microfluidic chip as it does not require sacrificing the model to complete assessment. These integrated sensors can therefore be used during the culturing phase to assess the successful establishment of continuous confluent monolayers prior to introduction of ALI or stimuli It is important to note that TEER on ALI cultures requires that the channel be filled with liquid prior to measurement. The effect of this intermediate transition between submerged and ALI conditions has not been extensively investigated.

#### 5.1.4. Inline Sensors

Maintaining environmental factors within acceptable ranges is essential to preserve cellular phenotypes; changes in pH, temperature, and gas compositions can have a significant effect on a culture’s health and, therefore, predictive value. As such, cultures are grown in strictly controlled incubators, culture medium is changed regularly, and care is taken to prevent significant environmental assaults. On the other hand, cells themselves can drive changes to their environment, for example secreting chemokines or decreasing their oxygen consumption rates in response to an administered stimulant. Tools with the ability to monitor these changes can offer valuable insights to researchers. Shaegh et al. employed low-cost opto-electronics to develop a real-time pH and oxygen concentration sensor suitable for real-time monitoring of these quantities in microfluidic chip effluents based on pH-indicator absorbance and fluorescence of an oxygen-sensitive probe [228]. Weltin et al. developed lactate concentration sensors by immobilizing lactate oxidase onto a hydrogel and coupling it with an electrode [229]. When lactate was generated in culture medium, it reacted with the enzyme to produce hydrogen peroxide; the hydrogen peroxide was then oxidized by the electrode, thereby transducing lactate production into an electrical signal [229]. Reviews regarding micro-scale optical and electrical sensors compatible with organ-on-chip-type platforms have been published by Grist et al. as well as Clarke et al. [230,231].

**Figure 7:**
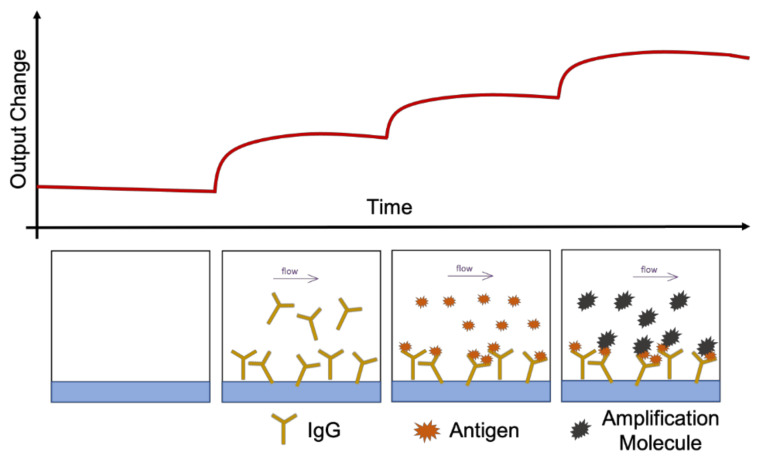
Schematic Illustration of Silicon Photonics Biosensing. As biomolecules bind to waveguides, the refractive index around the waveguide changes; these changes depend primarily on the molecular weight of the bound species and lead to changes in intensity measured by photodetectors.

Silicon photonic biosensors (Figure 7) may also find application in real-time and label-free sensing of secreted factors in microfluidic chip effluents. This emerging technology exploits changes in refractive index created by molecular interaction events, for example an antigen binding to an immobilized antibody. The operating principle of silicon photonic sensors involves infrared light travelling in raised nanometer scale silicon strips that can be lithographically formed onto substrates fabricated through the SOI platform. Light travelling in these strips, termed waveguides, creates an evanescent field in the surrounding space; when molecular interactions occur, such as an antibody binding to the surface of a waveguide, or an antigen binding to an antibody immobilized as such, this field is perturbed in a way that will influence the propagation of light in a measurable way [232]. Many applications currently rely on external sources of light (IR lasers) and photodetection (to measure the aforementioned perturbations), which can theoretically be integrated with a microfluidic chip upstream. An area of research emphasis in this field, however, is scaling down the laser and photodetection in cost and footprint [233], augmenting the potential applicability of this technology.

An example application relevant to the exposures discussed above might involve connecting a silicon photonic biosensor downstream of a microfluidic chip with anti-interleukin-8 antibodies preadsorbed onto the waveguide surface. Lung epithelial cells have been found to secrete IL-8 in lung inflammation [234], and exposure to cigarette smoke extract can increase IL-8 release as a function of concentration of the toxicant as well as the time after exposure [145]. Using an in-line sensor, researchers could then monitor, in real-time, secretion of IL-8 by cells into effluent after stimulation by cigarette smoke.

#### 5.1.5. Cellular Cross Talk

Transwell*^®^* and organ-on-chip technology can, also, enable interrogation of the interplay across multiple cell types. Osei et al., leveraging the former, demonstrated that co-culturing of epithelial cells with fibroblasts resulted in greater levels of inflammatory mediators (IL-8) and heat shock proteins (Hsp70) than in respective monocultures; fibroblasts incubated in medium conditioned by cigarette-smoke-extract-exposed airway epithelial cells (AEC), furthermore, released more IL-8 than those incubated in control AEC conditioned medium [150]. Microfluidic devices populated with multiple cell-types have also been reported (see Table 1); Barkal et al. employed a tri-culture model consisting of a cylindrical, HBEC-seeded airway in-between two endothelial-lined vessel structures, all within a fibroblast-encapsulated hydrogel to investigate pulmonary fungal infection [27]. This enabled measurement of integrated responses across the three cell types through analysis of media in the vessel compartments [27]. Park et al. also developed an epithelial–mesenchymal–endothelial airway-on-chip; utilizing separately cultured airway and vascular PDMS-based compartments, which could be coupled via plasma bonding, they were able to interrogate cross-talk between the different cell-types [28]. They found that, relative to a mono-culture airway epithelial ALI, contact with the vascular compartment (also containing fibroblasts) led to more robust airway epithelial differentiation (evidenced by the number of goblet and ciliated cells present) as well as increased barrier integrity (evidenced by greater TEER) [28]. These findings highlight the benefit of modular in-vitro models, where integrated responses can be compared with those of mono- or co-cultures to isolate the impacts of cross-talk. An alternative approach may involve comparing microfluidic airways-on-chip seeded with one cell type with an identical model seeded with another, or multiple, in parallel. For example, a model such as that developed by Sellgren et al., consisting of three stacked, membrane-separated microfluidic channels all housed within a PDMS device, could be seeded with airway-epithelial cells alone while replicates could be seeded with fibroblasts or endothelial cells [24]. Responses to cigarette smoke exposure could, then, be compared across the models housing the different cell types; these responses could, in turn, be compared to the complete tri-culture for isolation of cross-talk between the different cell-types.

### 5.2. Endpoint Analysis

Similar to traditional in vitro models, most tissues cultured within lung-on-a-chip systems are compatible with standard immunostaining techniques. Immunostaining can be performed on-chip or off-chip (i.e., on extracted hydrogels [22] to confirm the expression of characteristic biomarkers. On-chip assays minimize the chance of introducing artefacts during the extraction process, however off-chip assays benefit from using standardized staining techniques. For example, extracted cellular and extracellular components of a lung-on-a-chip device can be processed using histological readouts (e.g., Hematoxylin and eosin; Periodic Acid-Schiff stain) to characterize the epithelium and confirm pseudostratified morphology. Immunostaining methods can also be utilized to assess cell–cell interactions post exposure. For example, EMT can be detected through staining epithelial and mesenchymal markers and observing a transition of epithelial cells to mesenchymal phenotype through the decrease of epithelial markers (E-cadherin) and/or increase in mesenchymal markers [21,29].

As many lung-on-a-chip models focus on the alveolar–capillary interface, immunostaining has focused on protein complexes that indicate barrier formation. The cells of the epithelium form tight confluent monolayers that are separated by protein complexes, and their presence is often used as an identification of barrier formation. Tight junction proteins (occluding [26], zonula occludens [18,22,23,25,28,47,51]) and adherens junction proteins (E-cadherin [20,50,51]) can both be used to assess barrier formation. Similar to epithelial barriers, endothelial barriers can be confirmed through the presence of junctional proteins (e.g., VE-cadherin [20,26,50]). In the case of lung-on-a-chip models that focus on the airways, epithelial differentiation, and the composition of epithelial cells (i.e., number of goblet cells present) tend to be the focus of characterization. To confirm the model successfully establishes a pseudostratified epithelium, various markers of differentiation can be utilized. Mucin (i.e., MUC5AC [22,23,28] and MUC5B [24]) and cilia (i.e., Beta-Tubulin [23,24,28]) markers are most commonly used in the characterization of lung-on-a-chip devices. For devices containing smooth muscle cells or fibroblasts, the presence of alpha-smooth muscle actin positive cells can be used to confirm the presence or activation of myofibroblasts, and the orientation of the actin fibers can be used to assess cell alignment [22]. As myofibroblast accumulation and activation are a key hallmark of fibrosis, an increase in alpha smooth muscle expression can be used to assess a model’s ability to capture disease phenotypes [235].

## 6. Discussion

Lung-on-a-chip technologies are emerging as promising tools for drug discovery and disease modeling. However, there are areas in which improvement is possible, which would enable researchers to gain new insights into the underlying mechanisms of many respiratory diseases. In the lung, the interstitial space is a major site of pathology for respiratory diseases, but the mechanisms are not completely understood. Currently, most lung-on-a-chip models focus on the alveoli, precluding interrogation of mesenchymal cells and interstitial ECM. Further development of these models is required to be able to probe the biology and pathology essential for the discovery and development of novel therapeutics. A potential avenue for advancement of lung-on-a-chip technology is through the incorporation of cell embedded matrices that mimic the lung interstitium and can be patterned to model the luminal nature of the airway and microvasculature. These lumens can then be seeded with primary cells that facilitate the replication of epithelial and endothelial barrier functions. Lung-on-a-chip devices that reconstitute epithelial–stromal (ECM and vasculature) interfaces have the capability to expand the functionality of these devices for drug screening and disease modeling.

In contrast to standard monolayer cultures, where the cells are exposed to uniform conditions, organ-on-chip systems have cells exposed to different levels of stimulants or stressors. The 3D architecture permits creation of gradients in nutrients such as oxygen, or gradients of physical stresses. With the lung-on-chip technologies come the opportunity to extract tissue-level information, and subcellular and cellular information over time and space. The example of airway-on-chip technology being applied to the study of cigarette smoke, a significant and causal factor in the pathogenesis of chronic obstructive pulmonary disease, as well as electronic cigarettes, whose effects are not yet well-understood, highlights the potential value of this platform. Effects well-established in earlier studies on static ALI models or submerged monolayers were recapitulated in a dynamic ALI, while novel phenomena were observed in ciliary function. Compared to static ALI systems, the more realistic transport profile in a dynamic ALI can also inform design of drug delivery targeting strategies to optimize the aerosol size for improved deposition of the drug. We also believe there is the potential to augment the utility of these technologies with the integration of online and inline sensors for real time analysis as well as label-less microscopy methods enabling cultures of primary cells to be done so without modification or addition of fluorescent markers that could alter cellular phenotypes. The transition from endpoint analysis to continuous real-time analysis will provide the opportunity to gain further insight into the underlying mechanisms of biological interactions such as epithelial–mesenchymal transition and collagen remodeling.

### Hurdles to Translation and Remaining Challenges

Organ-on-chip systems show strong potential for improving preclinical-to-clinical translation during drug development [236,237]. There is increasing focus on using organ-on-a-chip technologies in the context of replacing animal studies during preclinical drug efficacy and safety testing [238]. While animal models can provide an entire in vivo system for testing drugs, the limitations of animal models, especially inter-species differences, render it difficult to predict translation between animals to humans. More human-relevant approaches include the generation of patient-derived organoids and organ-on-chip models, comprising patient-derived cells.

For respiratory diseases, drug development has an overall attrition near 70% [239]. The lack of efficacy during clinical trials and the poor representation of human disease in animal models is a particular challenge for respiratory diseases [240]. Here, lung-on-chip models that can reproduce the direct contact of epithelial cells with air will be essential for testing inhaled drugs, wherein the deposition of the inhaled drug formulation and the removal by mucociliary clearance can be studied. Furthermore, lung-on-chip models that permit cell–cell interactions, recreate perfusion and blood circulation for clearing drugs, and disease-specific characteristics such as the thick mucus and obstructed airway seen in COPD will be advantageous for generating more predictive data. Although the potential for precision or personalized therapy using lung-on-a-chip models have been shown through various approaches, the successful implementation of a personalized therapy for a patient with an airway or lung disease using lung-on-a-chip models has yet to be realized. Lung-on-a-chip models can achieve some level of personalization by using patient samples or by using biomarker levels typically seen in diseased patients, such as Benam et al.’s model that incorporated primary airway epithelium from COPD patients to show different cytokine release profile in response to inflammatory stimuli and cigarette smoke, and used controlled levels of interleukin-13 (IL-13) in healthy chips to induce aspects seen in asthma such as increased inflammatory cytokines excretion and reduced ciliary beating frequency of epithelial cells [23]. However, incorporation of other patient samples to “personalize” lung-on-a-chip models such as the use of human induced pluripotent stem cells (hiPSCs), or patient-specific ECMs have not been incorporated in lung-on-a-chip models although controllable integration of a decellularized ECM hydrogel has demonstrated the potential for this approach to individualized models [28]. Further levels of personalization on lung-on-a-chip models have yet to be realized as major challenges to successfully implementing precision medicine still exist including the ability of these models to reproduce multifactorial and chronic diseases, the acquisition of individual health data and the surrounding ethical issues, comparison of functional outputs from personalized lung-on-a-chip models with observed symptoms as well as responses to therapies and outcomes in patients, and the implementation of this technology in a feasible economic way [241]. As lung-on-a-chip, and many other organ-on-a-chip, technologies are still being developed, significant progress towards the translation and adoption of these organ-on-chip systems in the preclinical-to-clinical stages is also required to successfully bring personalized lung-on-a-chip models and precision medicine to fruition.

Organ-on-a-chip systems have the potential to have a large financial impact in pharmaceutical research and development, with average total cost reduction in the range of 10 to 26% [242] found in a survey of pharmaceutical and biotechnology companies, academia, and regulatory expertise. These cost reductions were based on organ-on-chips replacing some animal models and increasing predictability in the early phases, and in particular in lead optimization. However, large-scale adoption in organ-on-chip technologies has not yet been realized [243,244].

Some of the challenges have been related to cell sourcing, linking multiple organ-on-chip platforms, and agreement on what constitutes validation of the organ-on-chip models. One of the hurdles to wide adoption of organ-on-chip technologies is the gap between prototype devices and mass manufactured devices. There is no standardization for selecting the materials used to make microfluidic devices [245], and materials that lend themselves to rapid prototyping and small-scale fabrication such as PDMS are not suited for mass production and adoption in industry [246,247]. An ISO task force, ISO/CD 22,916 Laboratory equipment—Interoperability of microfluidic devices, has been established to develop standards for microfluidic interfaces, interconnects, bonding methods for materials, and other basic guidelines [248]. From the perspective of organ-on-chips, standards would strongly benefit inter-compatibility of different components within a system by having standard interconnects, testing for reliability, modularity, flow control, and assembly. Standardization of the platforms can facilitate industry adoption, so that companies do not need to invest in separate, non-compatible technologies, and will enable scale-up of media, compound, and sample delivery and extraction from parallelized operation of many organ-on-chip systems. Standardization of interconnects will be particularly important for lung-on-chip technologies, which require dynamic control of air flow as well as of liquid media perfusion.

Another major hurdle to translating organ-on-chips to industry is the use of different cell types among different organ-on-chip models [249]. While the organ-on-chip models can be used to predict drug responses for individuals or patient sub-populations, it can be challenging to obtain enough cells to establish sufficient microtissues for large scale studies. Sakolish et al. have found that the reproducibility of a human renal proximal tubule model depended greatly on the cell source and recommend that such systems should be developed in parallel with using a commercially available, well-characterized cell population for scalability [250]. The phenotypic characterization and quality control for either primary cells from patients or cells from induced pluripotent stem cell-derived cells will be essential for validation and large-scale deployment.

The Tissue Chip Testing Centers established in the United States aim to assess robustness, reproducibility, and reliability of separate organ-on-chip platforms as well as the challenges in transferring organ-on-chip systems from the initial developers to end-users. The U.S. National Institutes of Health also fund a Microphysiological Systems (MPS) database, a repository for technical, analytical, and biological data, which can be used to help establish standards for organ-on-chip validation [236]. The hesitation in industry may also be related to the early stage of technology development and lack of standard regulatory validation processes [244], or standardized methods for generating data from organs-on-chips. Continued engagement and collaboration between organ-on-chip developers, industry end-users, and regulatory bodies will facilitate adoption of this technology and contribute to the development of new treatments for respiratory diseases.

Other non-animal modeling approaches to study human lung biology and disease include computational, in silico methods. In silico modeling has been investigated to understand how airflow dynamics can affect particle deposition in the lungs, and predictive models will require not only an accurate model of the airway tree but also incorporation of the effects of the surfactant layer on the airway wall, among other parameters [251]. More generally, computational methods have been used to examine interactions between chemical compounds and biological targets [252]. Molecular modeling has been used to examine the interaction between small molecules and proteins in the absorption, distribution, metabolism, and excretion process. Data modeling involves quantitative structure–activity relationship or quantitative structure–property relationship studies [253]. Quantitative Systems Toxicology (QST) integrates computational and experimental methods: by using in vitro cell toxicity data and computational models of systems biology, the QST may contribute to predicting drug toxicity. Given the appropriate data quantity and data quality, machine learning approaches can be used throughout the drug discovery pipeline [254] to reduce attrition. Overall, in silico methods may complement in vitro organ-on-chip methods in drug discovery to predict toxicology, taking into account the genetic diversity in human populations [255]. Combined in silico and in vitro organ-on-chip methods may also permit data from multiple experiments and diverse organ-on-chip systems to be integrated.

## 7. Conclusions

Lung-on-a-chip platforms have shown promise for applications in drug development and inhalation assays. In this paper, we summarize existing lung-on-a-chip models, emphasizing important features of these models as well as looking at their suitability for studying airway remodeling and repair. To develop effective and functional models of the respiratory system there must be the understanding that there is no one size fits all model, and that in-depth knowledge of lung anatomy, histology, and function is critical for model development. The reproduction of key airway features, including the air–liquid interface and defined cellular architecture, are essential for being able to investigate the complex interplay between cells, ECM, and environment. Within these in vitro models, the stiffness of the ECM substitute should aim to mimic the in vivo counterpart.

Airways-on-chip have been used to explore the effects of exposures to clinically relevant substances. We reviewed various assays currently compatible with lung-on-a-chip technologies and highlighted emerging techniques that can be applied to further enhance the characterization capacity of lung-on-a-chip technologies. In future, multi-organ interactions, wherein multiple organs-on-chips are connected in a network, can permit more complete characterization of tissue response and development of precision medicine. The readouts from single lung-on-chips and eventually from multiple interconnected organs-on-chips, which to date have been related to cellular function, need to be linked to human clinical data. In this way, organs-on-chips based on patient-specific cells may be used to improve patient care and clinical outcomes. For this, further development of sophisticated sensors and microscopy techniques specifically designed for microfluidic lung-on-a-chip devices to further enhance the functionality of these devices as tools for disease modeling and drug discovery will be essential.

## Figures and Tables

**Table 1:** Cellular Make-Up of Current Lung-On-A-Chip Devices.

Area of Interest	Culture Type	Cells	Cell Origin	Tissue Interface	Air–Liquid Interface	Ref
Alveolus	Monoculture	Lung alveolar (type II) epithelial-like cells (A549)	Cell Line	Epithelium	No	[17]
Alveolus	Monoculture	Lung alveolar (type II) epithelial-like cells (A549)	Cell Line	Epithelium	No	[18]
Alveolus	Monoculture	Lung alveolar (type II) epithelial-like cells (A549)	Cell Line	Epithelium	Yes	[19]
Alveolus	Co-Culture	Human alveolar epithelial cells (hAECs) Human pulmonary microvascular endothelial cells	Primary	Alveolar– Capillary	Yes	[26]
Alveolus	Co-Culture	Bronchial epithelial cells (16 HBE14 o-)/Primary human pulmonary alveolar epithelial cells (pHPAECs) Human umbilical vein endothelial cells (pHUVEC)	Cell Lines/Primary	Alveolar– Capillary	Yes	[50]
Alveolus	Co-Culture	Lung alveolar (type II) epithelial-like cells (A549)/Murine alveolar epithelial cells (AECs)	Cell Lines/Primary	Alveolar– Capillary	Yes	[40]
Alveolus	Co-Culture	Human pulmonary alveolar epithelial cells (HPAEpiCs) Human umbilical vein endothelial cells (HUVEC)	Cell Lines	Alveolar– Capillary	No	[20]
Alveolus	Co-Culture	Human alveolar epithelial cells Human lung microvascular endothelial cells	Primary	Alveolar–Capillary	Yes	[47]
Alveoli	Monoculture	Human alveolar epithelial cells (hAECs)	Primary	Epithelium	Yes	[25]
Alveoli	Co-Culture	Human bronchial epithelial cells (BEAS-2 B) Human vascular endothelial cells (HUVECs)	Cell Lines	Alveolar–Capillary	Yes	[21]
Alveoli	Co-Culture	Human alveolar epithelial cells (hAECs) Human lung endothelial cells	Primary	Alveolar–Capillary	Yes	[51]
Airway	Co-Culture	Human airway epithelial cells (hAECs) Human pulmonary microvascular endothelial cells	Primary	Epithelial– Endothelial	Yes	[23]
Airway	Co-Culture	Human airway epithelial cells (Calu-3) Human bronchial smooth muscle cells (hBSMC)	Cell Lines	Epithelial–Mesenchymal	Yes	[22]
Airway	Tri-Culture	Human tracheo-bronchial epithelial cells Human lung fibroblasts Human lung microvascular endothelial cells	Primary	Epithelial–Stromal/Vascular	Yes	[24]
Airway	Tri-Culture	Human tracheal epithelial cells Human Lung fibroblasts Human dermal microvascular endothelial cells	Primary	Epithelial–Stromal/Vascular	Yes	[28]
Airway	Tri-Culture	Human bronchial epithelial cells Normal pulmonary fibroblasts Human lung microvascular endothelial cells	Primary	Epithelial–Stromal/Vascular	Yes	[27]

**Table 2:** ECM Dimensionality and Composition of Current Lung-On-A-Chip Devices.

Dimension	ECM Substitute Type	ECM Substitution Material	Lung ECM Replicated	Area of Focus	Reference
2	Non-Coated Membrane	Polyester	Basement Membrane	Airway	[101]
2	Non-Coated Membrane	PET	Basement Membrane	Alveolus	[19]
2	ECM Coated Membrane	PDMS	Basement Membrane	Alveolus	[40]
2	ECM Coated Membrane	PDMS	Basement Membrane	Alveolus	[108]
2	ECM Coated Membrane	PDMS	Basement Membrane	Alveolus	[24]
2	ECM Coated Membrane	Polyester	Basement Membrane	Airway	[50]
2–2.5	ECM Coated Membrane Combined With Additional Channel	PTFE and PET	Interstitial Layer	Airway	[23]
2.5	ECM Thin Film	Collagen and Elastin	Basement Membrane	Alveoli	[27,69]
2.5	ECM Thin Films	Collagen I and Matrigel	Basement Membrane	Airway	[27,69]
2.5–3	ECM Hydrogel	Collagen and Matrigel	Interstitial Matrix	Airway	[22]
3	ECM Hydrogel	Collagen and Fibrinogen	Interstitial Matrix	Airway	[28]
3	ECM Hydrogel	Decellularized ECM	Interstitial Matrix	Airway	[51]
3	ECM Hydrogel	GelMA	Interstitial Matrix	Alveoli	[25]

**Table 3:** Summary of Cigarette Smoke Extract Preparation Methods.

Reference	CSE Concentration	Solvent	Notes
Hoshino et al. [135]	0.5 filtered cigarettes/mL	DMEM	CSE 0.22 micron filtered [nicotine] determined via HPLC to be 1 mg/mL Prepared immediately prior to use
Carnevali et al. [139]	0.04 filterless cigarettes/mL	DMEM	CSE 0.2 micron filtered Used within 30 min
Heijink et al. [140]	0.08 filterless reference cigarettes/mL	EMEM	CSE 0.22 micron filtered Used within 30 min
Richter et al. [146]	0.04 filterless reference cigarettes/mL	RPMI 1640	CSE 0.22 micron filtered Used immediately
Kode et al. [148]	0.1 reference cigarettes/mL	DMEM, RPMI 1640	CSE 0.45 micron filtered OD of 0.74 measured at 320 nm Used immediately
Witherden et al. [147]	1 filterless cigarette/mL	LPHM	Freshly generated for each experiment

**Table 4:** Summary of Solution-Based Electronic Cigarette Aerosol Studies.

Reference	Cells	eCVE Preparation	Results
Romegna et al. [161]	BALB/3T3 fibroblasts (mouse)	200 mg e-liquid extracted into 20 mL culture medium	One of 21 extracts was cytotoxic (51% viability) at highest (undiluted concentration); all others not cytotoxic
Cervellati et al. [158]	A549	Whole smoke delivered to incubator with lids of culture plate removed	Flavored e-liquids and e-liquids with nicotine led to decreased in viability (LDH)
Higham et al. [157]	Neutrophils isolated from peripheral donor blood	50–300 mL aerosol bubbled through RPMI 1640 culture medium (volume unspecified). Normalized to OD at 320 nm in culture medium. 0.22 micron filtered	Increase in MMP-9 and IL-8 release with exposure to eCVE
Putzhammer et al. [159]	HUVEC	88.5 mg liquid (equivalent to 700 mL aerosol) extracted into 8 mL culture medium. 0.2 micron filtered. Prepared freshly prior to experiments	Some e-liquid aerosols decreased viability, one of 11 tested increased oxidative stress. Results were liquid dependent. The same electronic cigarettes were used with different liquids; authors isolated effects to liquids.
Taylor et al. [162]	NCI-H292	550 mL aerosol bubbled through 20 mL DMEM/F12. Nicotine content characterized by GC-MS, tar by OD at 320 nm	No cytotoxic effects or oxidative stress induced by eCVE
Leslie et al. [156]	BEAS-2 B, IB3-1, C38 (human bronchial epithelium cell lines), Wi-38 fibroblasts, J774 THP-1 macrophages	490 mL aerosol extracted into 10 mL DMEM/F12, EMEM, or RPMI 1640. Used within 1 h	Some extracts reduced viability below 70% (considered cytotoxic), varied depending on flavor and cell line
Taylor et al. [163]	HUVEC	550 mL aerosol extracted into 20 mL Vasculife culture medium. Nicotine concentration qualified via GC-MS	eCVE did not inhibit endothelial cell migration
Bengalli et al. [160]	A549, NCI H441	11 L aerosol extracted into 25 mL OPTIMEM culture medium, 0.2 micron filtered and frozen at −20 until use	Viability and barrier integrity decreased with exposure to certain flavors, one of which led to an increase in IL-8 and MCP-1 release. Unflavored liquids produced insignificant effects. Nicotine was not found to be a factor.
Higham et al. [136]	Calu-3, primary bronchial epithelial cells (from healthy and COPD patients). Cultured at ALI but exposed to liquid solution.	50–300 mL aerosol bubbled through DMEM/F12 culture medium (volume unspecified). Normalized to OD at 320 nm in culture medium. 0.22 micron filtered	eCVE had cytotoxic effects and increased IL-6 and IL-8 while decreasing TEER, indicating a decrease in barrier integrity

**Table 5:** Summary of Electronic Cigarette Aerosol Studies at Static Air–Liquid Interface.

Reference	Exposure System	Cells	Findings
Scheffler et al. 2015 a [187]	CULTEX^®^	Primary BECs	Higher oxidative stress and decreased viability found
Scheffler et al. 2015 b [188]	CULTEX^®^	PBECs, CL-1548, A549	Decrease in viability, with sensitivity dependent on cell line
Lerner et al. [189]	Custom configuration	NCI-H292, BEAS-2 B	Increased IL-6 and IL-8 secretion
Iskandar et al. [186]	VITROCELL^®^	Primary BECs	Viability and ciliary beating unchanged, IL-8 increased
Antherieu et al. [190]	VITROCELL^®^	BEAS-2 B	Viability and oxidative stress levels unchanged, modest increase in IL-6
Hwang et al. [191]	Custom configuration	A549	Necrotic cell death induced, host defense decreased
Herr et al. [192]	Custom configuration	Primary BECs, Calu-3, HCI-H292	IL-8 secretion increased; no significant change in barrier integrity
Bathrinarayana et al. [174]	Custom configuration	CALU-3 (HBEC cell line) with MRC-5 (human pulmonary fibroblast cell line) in co-culture	ECA decreased viability at exposure times greater than three hours, increased IL-6 and IL-8 secretion, and increased oxidative stress (via hydrogen-peroxide assay)
Ghosh et al. [175]	Custom configuration	Primary human BECs	Upon exposure to ECA barrier integrity (as assessed by TEER and permeability to FITC-dextran) decreased, ciliary beat frequency was reduced, E-cadherin expression insignificantly affected
Ganguli et al. [193]	Custom configuration	Primary human BECs, NCI-H441 (human alveolar EC line)	Aerosolization wattage positively correlated with concentration of ECA particulates and presence of nicotine in e-liquid; transcription of genes related to oxidative-stress, inflammatory mediator expression (MMP-1, interleukins 1 B, 6, 8, 10) and depended on e-liquid flavor
